# Sensitivity of *Zea mays* and Soil Microorganisms to the Toxic Effect of Chromium (VI)

**DOI:** 10.3390/ijms24010178

**Published:** 2022-12-22

**Authors:** Jadwiga Wyszkowska, Agata Borowik, Magdalena Zaborowska, Jan Kucharski

**Affiliations:** Department of Soil Science and Microbiology, Faculty of Agriculture and Forestry, University of Warmia and Mazury in Olsztyn, 10-727 Olsztyn, Poland

**Keywords:** chrome translocation, chromium bioaccumulation, genetic diversity, ecophysiological diversity, bacteria, fungi

## Abstract

Chromium is used in many settings, and hence, it can easily enter the natural environment. It exists in several oxidation states. In soil, depending on its oxidation-reduction potential, it can occur in bivalent, trivalent or hexavalent forms. Hexavalent chromium compounds are cancerogenic to humans. The aim of this study was to determine the effect of Cr(VI) on the structure of bacteria and fungi in soil, to find out how this effect is modified by humic acids and to determine the response of *Zea mays* to this form of chromium. A pot experiment was conducted to answer the above questions. *Zea mays* was sown in natural soil and soil polluted with Cr(VI) in an amount of 60 mg kg^−1^ d.m. Both soils were treated with humic acids in the form of HumiAgra preparation. The ecophysiological and genetic diversity of bacteria and fungi was assayed in soil under maize (not sown with *Zea mays*). In addition, the following were determined: yield of maize, greenness index, index of tolerance to chromium, translocation index and accumulation of chromium in the plant. It has been determined that Cr(VI) significantly distorts the growth and development of *Zea mays*, while humic acids completely neutralize its toxic effect on the plant. This element had an adverse effect on the development of bacteria of the genera *Cellulosimicrobium*, *Kaistobacter*, *Rhodanobacter*, *Rhodoplanes* and *Nocardioides* and fungi of the genera *Chaetomium and Humicola*. Soil contamination with Cr(VI) significantly diminished the genetic diversity and richness of bacteria and the ecophysiological diversity of fungi. The negative impact of Cr(VI) on the diversity of bacteria and fungi was mollified by *Zea mays* and the application of humic acids.

## 1. Introduction

The presence of heavy metals in the natural environment, including the atmosphere, pedosphere and hydrosphere, is a global problem [1,2,3] which poses a threat to the proper growth of all organisms [4,5,6]. There are over 10 million contaminated sites in the world, covering over 20 million hectares of land, of which over 50% are polluted with heavy metals [7,8]. These pose a serious threat to the environment and the ecological safety of the human population; additionally, they harm the agricultural productivity of soils [9,10,11]. Chromium is a heavy metal that is widespread in the environment [1,2,12]; it is an element that is present naturally in the earth’s crust [5]. It exists in various oxidation states, from −2 to +6 [5,13]. It also exists in various complex ions, such as CrOH^2+^, CrO_4_^2−^ and CrO_3_^3−^ [13]. Compounds of trivalent and hexavalent chromium are particularly long-lasting [5,14]. Transformations of chromium compounds in soil are among most complex due to the different oxidation states in which this element appears [15,16], depending on the chemical and physical properties of the soil [17]. The oxidation state of chromium is also affected by the soil reaction, its grain-size distribution, redox potential and content of humus [18,19,20], as well as the presence of other metals. For example, Fe^2+^ contributes to the reduction of hexavalent chromium and Mn^6^ to the oxidation of trivalent chromium [21,22,23]. According to Xu et al. [21] the change in Cr(VI) content in the soil results from the competition between Cr(III) oxidation and Cr(VI) reduction.

Chromium is classified as an element with low migration activity in soils and lower ability—compared with other elements—to form lasting complexes with organic compounds [8,19,24]. The potential bioavailability of chromium compounds depends mainly on its oxidation state, the adsorption ability on the surface of soil minerals, the organic matter content and the presence of microorganisms [18,19,20]. Cr(III) compounds are easily adsorbed by soil colloids, whereas Cr(VI) is rapidly oxidized and highly soluble and does not undergo biodegradation [15,17,25]. Cr(III) compounds in quantities found naturally in the environment are not toxic because they are characterized by lower solubility and bioavailability to plants and microorganisms than Cr(VI) compounds [26,27]. Cr(III) is absorbed passively by plants, while Cr(VI) is actively absorbed [17,28]. In response to Cr(VI) pressure, plants activate several mechanisms and tolerance strategies that limit the negative impact of metals. One of them is the change in gene expression and protein function modulation, which is to ensure the maintenance of homeostasis of metabolic processes in the plant [29,30]. It has also been hypothesized that the uptake of Cr by roots relies on the HPO_4_^2−^ or SO_4_^2^ carriers, which explains why ions of sulfates, nitrates, phosphates and iron, by competition, inhibit the accumulation of Cr(VI) [31]. The sequestration and detoxication of heavy metals in plants are also mediated by glutathione (GSH) and metallothionein (MT), the precursor of which is cysteine [29]. Moreover, cysteine participates in the alleviation of the toxic effects of Cr(VI) by reducing the peroxidation of lipids [32].

Chromium enters the natural environment as a consequence of air and water erosion of rocks [33]. Natural sources of air pollution with chromium are forest fires and volcanic eruptions, whereas anthropogenic sources include combustion processes and industrial emissions [34,35]. The largest amounts of this metal enter the environment through anthropogenic activities [36,37]. The main sources are: the mining of chromium ores; the metallurgical industry, i.e., the production of iron, nickel and cobalt alloys; the chemical industry, i.e., the production of catalysts, paints, textiles, fungicides, products used for wood preservation, corrosion inhibitors and battery cells; and the tanning industry [33,37,38]. From an ecological perspective, it is extremely dangerous when chromium accumulates in soil, as soil is the main link in the natural cycle of chemical elements and the first component in the trophic chain: soil–plant–animal–man [7,38,39]. Any distortion of the balance between these components can have consequences in the other components in the system [5,13,40]. Chromium enters the food chain mainly via plants, posing a threat to the health of animals and people [7]. According to the International Agency for Research on Cancer, Cr(VI) compounds are classified in Group 1, i.e., highly carcinogenic substances, [41]. The Agency for Toxic Substances and Disease Registry placed Cr(VI) in the 17th place in a list of substances posing the most severe threat to human health [42]. According to Rahman and Singh [2] and Arshad et al. [43], Cr(VI) is characterized by very high toxicity, i.e., from 100- to 1000-fold more than that of Cr(III) [14,34,44]. Its toxicity is only surpassed by arsenic (As), lead (Pb), mercury(Hg) and cadmium (Cd) [2,42].

In view of the severe risk to the proper functioning of all organisms due to soil contamination with chromium, intensive investigations have been conducted in recent years in order to develop remediation methods that are financially viable, easy to implement, stable, acceptable by society and environmentally friendly, and which will not lead to any disturbances in soil fertility and biodiversity [37,45,46,47]. Such methods include microbiological remediation, bioaugmentation, and biostimulation [15,37,48,49,50]. The toxicity of Cr(VI) means that very few microorganisms in the soil are tolerant to it. However, some microorganisms (*Aeromonas*, *Escherichia*, *Pseudomonas*, *Enterobacter*, *Pannonibacter* and *Oscillatoria*) can perform the enzymatic reduction of this element [37,45,51]. As such, biostimulation methods are based on the stimulation of indigenous microorganisms exhibiting Cr(VI)-reducing capabilities in soil [52]. In comparison with bioaugmentation, biostimulation is less invasive to the environment, more rapid, as it does not require screening tests or acclimatization, and able to limit the scale of disturbances to the microbial assemblages in soil [37]. Restoring the biological equilibrium in soils contaminated with chromium will make it possible to regain the productivity of these soils and ensure food security [7,53].

The above considerations encouraged us to conduct a study aiming to determine the effect of Cr(VI) on the structure of bacteria and fungi in soil and to determine the possible contributions of *Zea mays* cultivation and the application of humic acids in restoring the soil microbiological balance. This study will also make it possible to predict the responses of soil microbial communities and plants to such contamination. Moreover, thorough knowledge of the mechanisms through which Cr(VI) affects the biological features of soil will allow us to identify the best ways to detoxify soils polluted with this element, which, in turn, will result in the improved health of soils.

## 2. Results

### 2.1. Zea mays Reaction to Soil Contamination with Cr(VI)

Chromium is an element that plants do not need, and Cr(VI) added to soil in a dose of 60 mg kg^−1^ d.m. of soil (Table 1, Figure S1) proved to be highly toxic to *Zea mays*. It caused a decrease in the yield of aerial parts by 90% and that of roots by 92%. It also decreased plant height by 49% and lowered the greenness index by 47% and 28% in the Biologische Bundesanstalt, Bundessortenamt and Chemical (BBCH) Scale (14 and 19, respectively). The adverse impact of Cr(VI) was completely alleviated by soil treatment with HumiAgra.

In the chromium-polluted group, there was significantly more of this element in both aerial parts and roots of *Zea mays* (Table 2). Significantly less of this element was found in all parts of the plants fertilized with HumiAgra. The content of chromium in aerial parts, irrespective of HumiAgra fertilization, ranged from 0.66 to 1.04 mg kg^−1^ d.m., and in the roots from 1.23 to 17.67 mg kg^−1^ d.m. Obviously, higher concentrations were noted in *Zea mays* grown in chromium-polluted soil, and the lower ones in unpolluted soil.

The data presented above had a significant influence on the values of the BF and TF indices (Table 3). They indicated higher bioaccumulation of chromium in roots (BF_R_) than in aerial parts (BF_A_). The total bioaccumulation of chromium in *Zea mays* was relatively small, as suggested by the BF values.

In a maize plant not polluted with chromium, the BF values ranged from 0.100 to 0.265, while in the ones polluted with this element, this index varied from 0.117 to 0.258. Lower values of the BF index were observed in *Zea mays* grown in soil fertilized with HumiAgra than in maize plants cultivated in soil not treated with this preparation. Low values of the TF index (0.059–0.119) in *Zea mays* grown in Cr(VI) polluted soil provide further evidence for the dominant concentration of chromium in roots rather than in the aerial organs of this plant. Regardless of the values of these indices, the TI index (0.940) proves that the test plant was highly tolerant to chromium contamination when grown in soil fertilized with HumiAgra.

### 2.2. Reaction of Bacteria and Fungi to Soil Contamination with Cr(VI)

#### 2.2.1. Breeding Microorganisms

**Figure 1:**
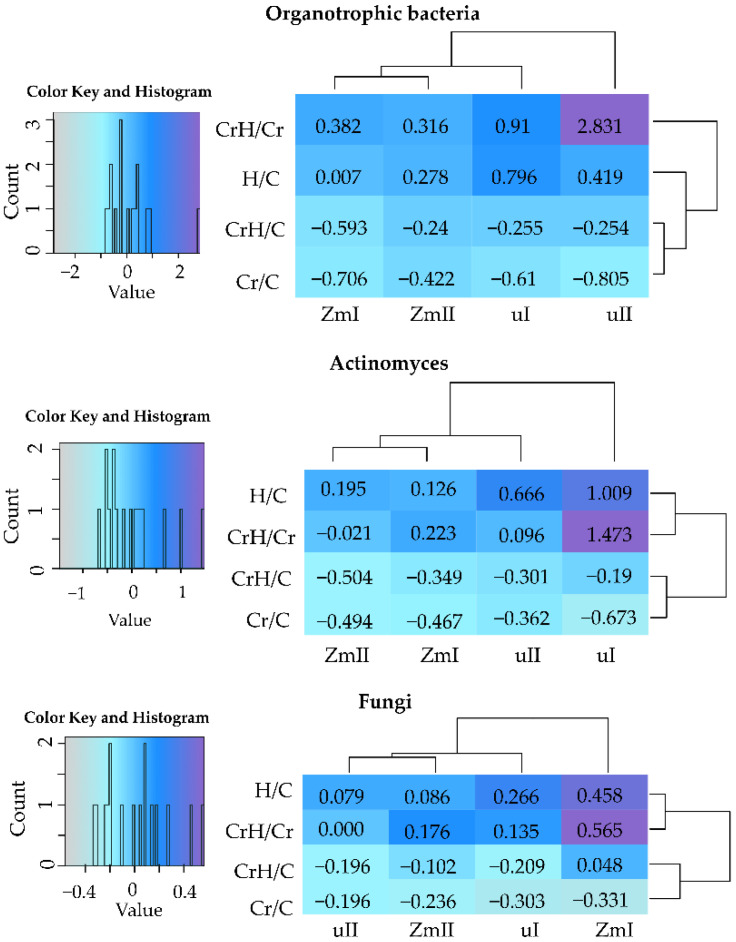
Index of the influence (IF) of Cr (VI) and HumiAgra (H) on the number of microorganisms. C—unpolluted unsown soil; C—soil polluted with Cr(VI); H—soil with the addition of HumiAgra; u—unsown soil; Zm—soil sown with *Zea mays*; I—25th day of research, II—50th day of research.

The abundance of microorganisms in soil was modified by Cr(VI), HumiAgra and the cultivation of *Zea mays*. The impact of chromium on organotrophic bacteria, actinomycetes and fungi was significantly negative, while the influence of HumiAgra and *Zea mays* on these microorganisms was positive (Table S1). Negative values of IF_Cr(VI)_ were observed for all microorganisms, regardless of the date of determination and soil use (Figure 1).

**Figure 2:**
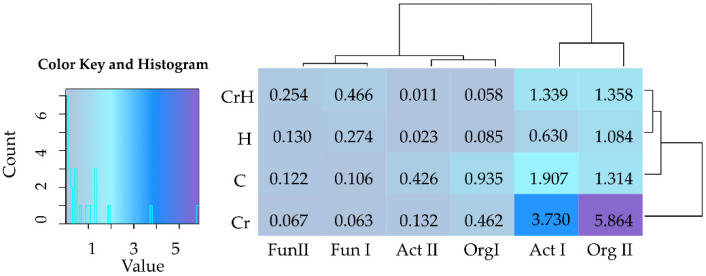
Index of the influence of maize (IF_Zm_) on the number of microorganisms. C—unpolluted unsown soil; Cr—soil polluted with Cr(VI); H—soil with the addition of HumiAgra; Zm—soil sown with *Zea mays*; I—25th day of the research, II—50th day of the research.

Nevertheless, these values prove that fungi were more tolerant to the negative effect of this element than organotrophic bacteria and actinomycetes. In contrast to Cr(VI), the effect of HumiAgra (IF_H_) on the analyzed microorganisms was positive. This index, on any test day of the experiment, achieved positive values, and its value was greater whenever the contribution of H to the shaping of counts of microorganisms was larger. The positive IF values noted in the CrH/Cr variable and its higher values in the Cr/C variable prove that HumiAgra reduced the adverse effect of Cr/C on the analyzed groups of microorganisms. The positive influence on soil microorganisms was also affected by *Zea mays* (Figure 2). The IF_Zm_ values were positive in all experimental objects. The IF_Zm_ value on day 50 of the experiment implies that *Zea mays*, through its root system, could more effectively stimulate the multiplication of organotrophic bacteria than actinomycetes or fungi.

The colony development index (CD) of microorganisms describes changes in the succession induced by the tested factors. A CD higher than that determined in the control indicates an increased multiplication of fast-growing (strategy K) microorganisms, while a lower CD indicates the prevalence of slowly reproducing (strategy r) microorganisms.

The data collated in Table S2 show that soil contamination with Cr(VI) intensified the development of strategy K fungi, regardless of the application of HumiAgra, whereas in the series with this preparation, it stimulated the multiplication of strategy r organotrophic bacteria. The multiplication of strategy K fungi induced by the presence of Cr(VI) was accompanied by their decreased ecophysiological diversity (Table S3). The latter was evidenced by the decreased value of the EP index.

##### Bacteria

**Figure 3:**
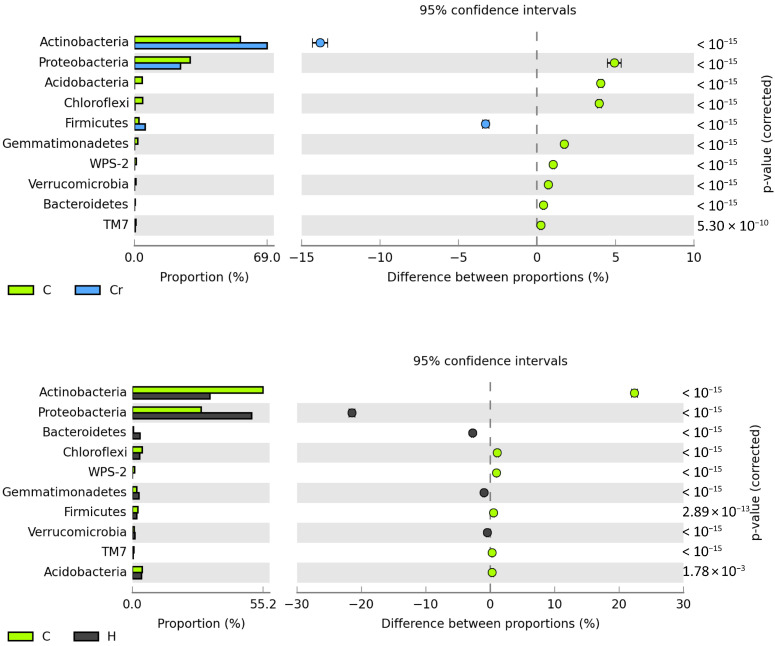

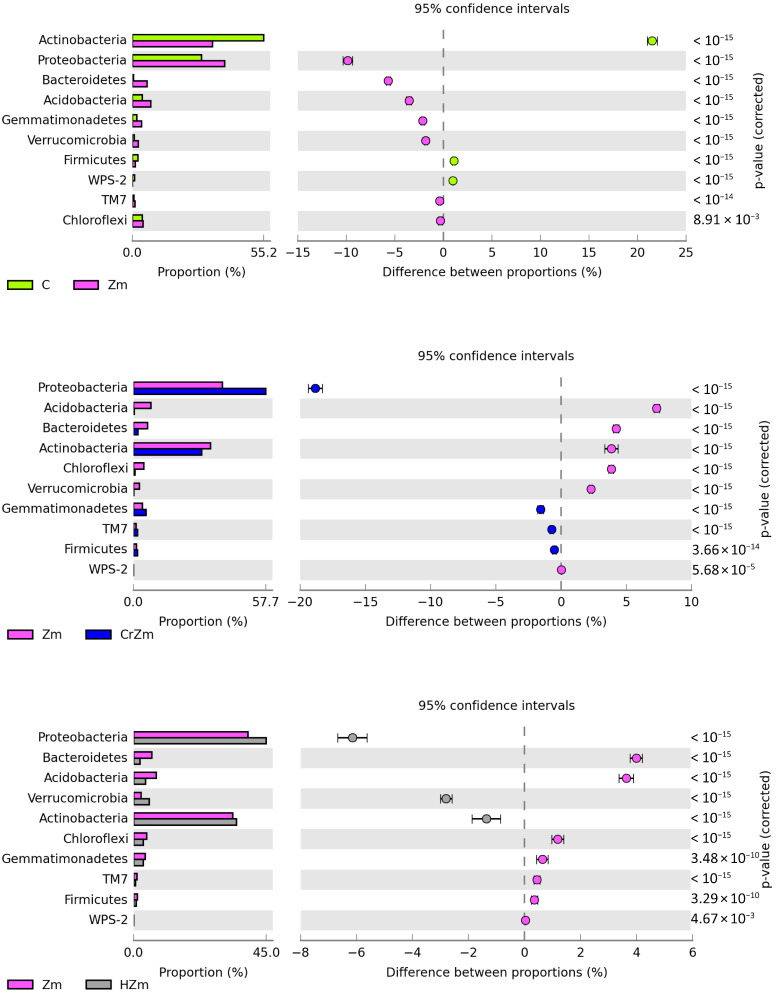
Effect of Cr(VI), *Zea mays* and HumiAgra on the relative abundance of the dominant types of bacteria. C—unpolluted unsown soil; Cr—soil polluted with chromium (VI); H—soil with the addition of HumiAgra; Zm—soil sown with *Zea mays*.

The tested soil contained the most numerous populations of bacteria from the phyla Proteobacteria and Actinobacteria (Figure 3).

The average percentage of *Proteobacteria* in the structure of microorganisms, irrespective of the analyzed objects, was 42.87%, whereas that of *Actinobacteria* equaled 39.24%. Likewise, the biggest changes caused by the independent variables were determined among bacteria of these two phyla. Chromium in soil unsown with *Zea mays* decreased the number of *Proteobacteria* by 4.93% and increased that of *Actinobacteria* by 13.82%, whereas HumiAgra exerted the opposite effect, i.e., it raised the share of bacteria of the phylum *Proteobacteria* by 21.46% and lowered that of *Actinobacteria* by 22.38%. Sowing with *Zea mays* contributed to an increase in the numbers of *Proteobacteria* by 9.84%, Bacteroidetes by 4.23% and *Acidobacteria* by 3.52% while decreasing that of actinobacteria by 21.53%.

In the soil polluted with Cr(VI) and cropped with *Zea mays*, the bacteria count of the phylum proteobacteria was increased by 18.82%, whereas those of *Acidobacteria*, *Bacteroidetes, Actinobacteria* and *Chloroflexi* were decreased by 7.34%, 4.23%, 3.86% and 3.85%, respectively. In general, it was possible to observe the unambiguously negative impact of Cr(VI), regardless of the type of land use, on bacteria of the phyla *Acidobacteria* and *Chloroflexi.* The decrease in the share of bacteria of the former ranged from 4.06% to 7.34%, and that of the latter from 3.85% to 3.98%. In turn, the cultivation of *Zea mays* on soil fertilized with HumiAgra resulted in an increase in the share of bacteria of the phylum *Proteobacteria* by 6.14% and a decrease in *Bacteroidetes* and *Acidiobacteria* by 3.99% and 3.64%, respectively.

**Figure 4:**
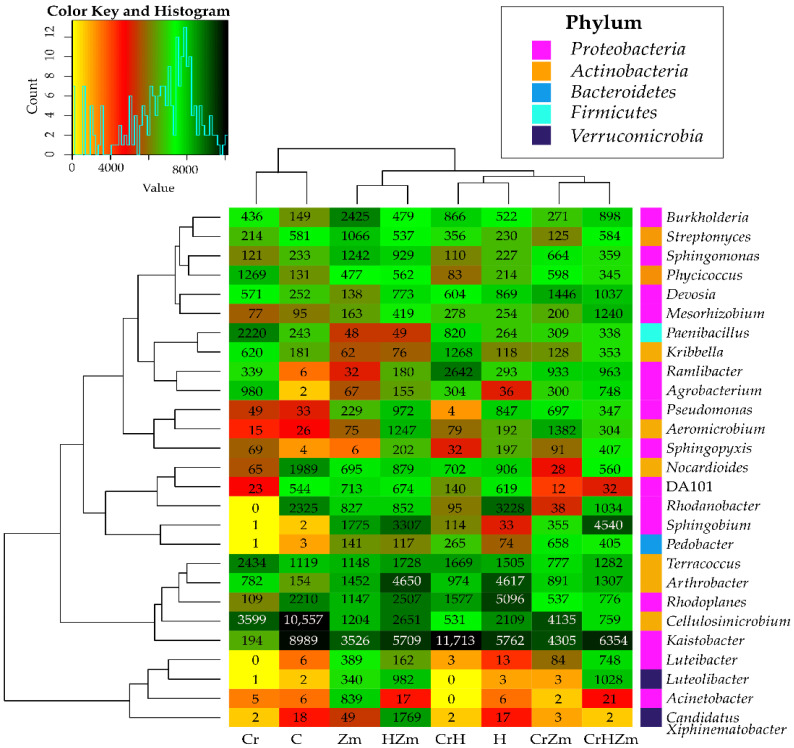
Effect of Cr(VI), *Zea mays* and HumiAgra on the relative abundance of dominant bacterial genus. C—unpolluted unsown soil; Cr—soil polluted with chromium (VI); H—soil with the addition of HumiAgra; Zm—soil sown with *Zea mays*.

On average, regardless of the experimental setting, bacteria of the genus *Kaistobacter* (5819 OTU) were the most numerous among the bacteria of the phylum Proteobacteria, followed by *Rhodoplanes* (1745 OTU), *Sphingobium* (1266 OTU) and *Rhodanobacter* (1050 OTU) (Figure 4). The least numerous bacteria of this phylum belonged to the genera *Acinetobacter* (112 OTU), *Sphinopyxis* (126 OTU) and *Luteobacter* (176 OTU). With regard the phylum *Actinobacteria*, the dominant genera were *Cellulosimicrobium* (3193 OTU), *Arthrobacter* (1853 OTU) and *Terracoccus* (1458 OTU), while the least abundant were the genera *Kribbella* (351 OTU), *Aeromicrobium* (415 OTU), *Phycicoccus* (460 OTU) and *Streptomyces* (462 OTU). Genera of the bacteria that belonged to remaining phyla were less numerous. Their average OTU number ranged from 208 (*Peddobacter*) to 536 (*Peanibacillus*).

In the bare, chromium-polluted soil, the most numerous bacteria were from the genera *Cellulosimicrobium*, *Kaistobacter*, *Rhodanobacter*, *Rhodoplanes* and *Nocardioides*. Soil contamination with Cr(VI) significantly decreased the counts of these genera. In turn, bacteria of the genera *Terracoccus*, *Paenibacillus*, *Phyciococcus*, *Agrobacterium*, *Arthrobacter*, *Kribbela*, *Devosia*, *Burkholderia* and *Ramibacter* found better conditions for their development in soil polluted with this element. Fertilization with HumiAgra contributed to changes in the counts of some bacterial genera. This preparation applied to soil not polluted with Cr(VI) caused an increase in the abundance of the bacteria *Rhodoplanes*, *Arthrobacter*, *Rhodanobacter, Terracoccus, Devosia* and *Pseudomonas* while decreasing the counts of *Kaistobacter*, *Cellulosimicrobium* and *Nocardioides*. The cultivation of *Zea mays* also modified the development of some genera of soil bacteria. This plant had a positive effect on the genera *Burkholderia*, *Sphinghobium, Arthrobacter, Sphingomonas, Streptomyces* and *Acinetobacter*, while having an adverse effect on *Kaistobacter, Cellulosimicrobium, Rhodoplanes* and *Rhodanobacter*. Soil pollution with Cr(VI) caused changes in the abundance of some genera of bacteria dwelling in soil under maize. It stimulated the multiplication of *Kaistobacter*, *Celllosimicrobium*, *Devosia, Aeromicrobium*, *Ramilibacter*, *Pseudomonas* and *Pedobacter,* while inhibiting the development of *Arthrobacter*, *Terracoccus*, *Sphingomonas*, *Rhodoplanes*, *Sphingobium*, *Burkhokderia* and *Streptomyces*. While Cr(VI) had a positive effect on some bacterial genera and a negative one on others, HumiAgra applied to soil cropped with *Zea mays* positively affected the vast majority of the bacterial genera. This was evidenced by the highest total number of OTU (32,584) obtained in the ZmH object.

**Figure 5:**
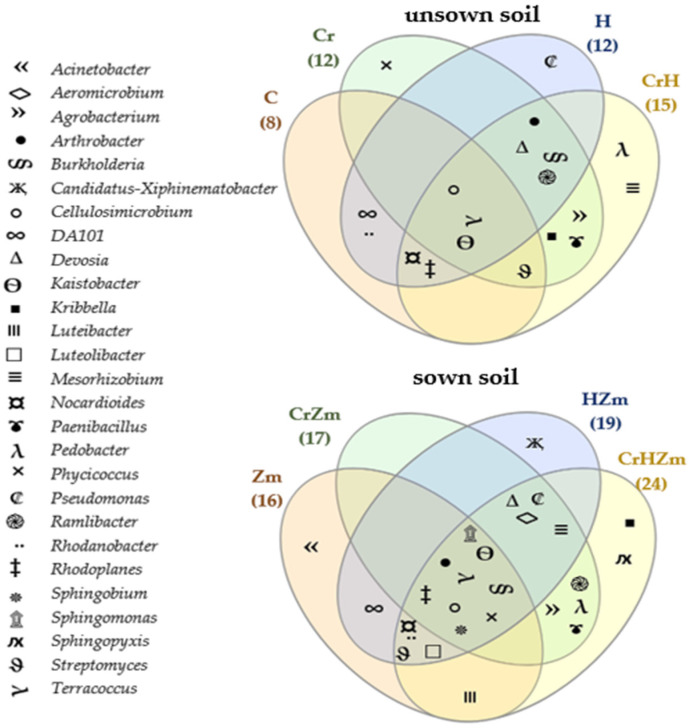
Venn diagram showing unique and common types of bacteria in unsown and sown *Zea mays* soil. C—unpolluted unsown soil; Cr—soil polluted with chromium (VI); H—soil with the addition of HumiAgra, CrZm—soil polluted with chromium and sown with *Zea mays* (VI); HZm—soil sown with *Zea mays* with HumiAgra.

The composition of the key bacterial assemblages, which create the core and unique bacteriome, was identified (Figure 5).

*Kaistobacter*, *Terracoccus* and *Cellulosimicrobium* were omnipresent in the core bacteriome in both soil cropped and uncropped with maize, irrespective of the soil pollution with Cr(VI) or its fertilization with HumiAgra. The core bacteriome in soil sown with maize was richer in *Sphingobium*, *Arthrobacter*, *Burkholderia*, *Rhodoplanes*, *Sphingomonas* and *Phycicoccus*, in addition to the genera mentioned above. The higher number of bacterial genera composing the core bacteriome in the soil under maize evidences the greater stability of bacteria on the genus level in the cropped soil.

**Figure 6:**
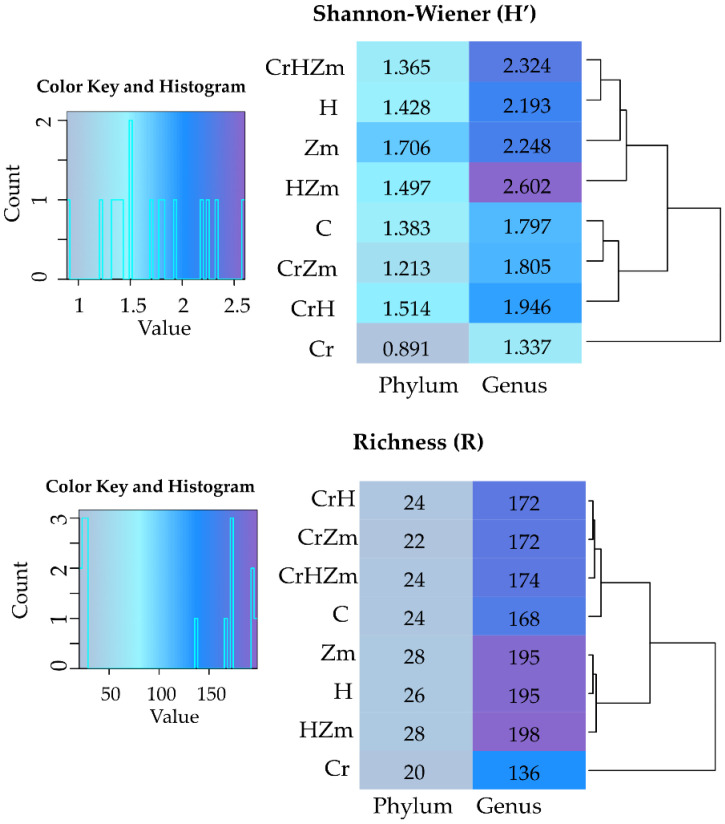
Shannon-Wiener index (H‘) and the richness (R) of bacteria in the soil. C—unpolluted unsown soil; Cr—soil polluted with Cr(VI); H—soil with the addition of HumiAgra; u—unsown soil; Zm—the soil sown with *Zea mays*.

The contamination of soil with Cr(VI) significantly decreased the diversity and richness of bacteria, irrespective of the soil use, whereas the HumiAgra added to soil unsown with maize caused an increase in the diversity of both phyla and genera and in the richness of genera (Figure 6). Cultivation of *Zea mays* significantly increased the diversity of both phyla and genera as well as the richness of bacteria. The adverse effect of Cr(VI) on the diversity and richness of bacteria was alleviated by the application of HumiAgra to soil (CrHZm variant).

##### Fungi

**Figure 7:**
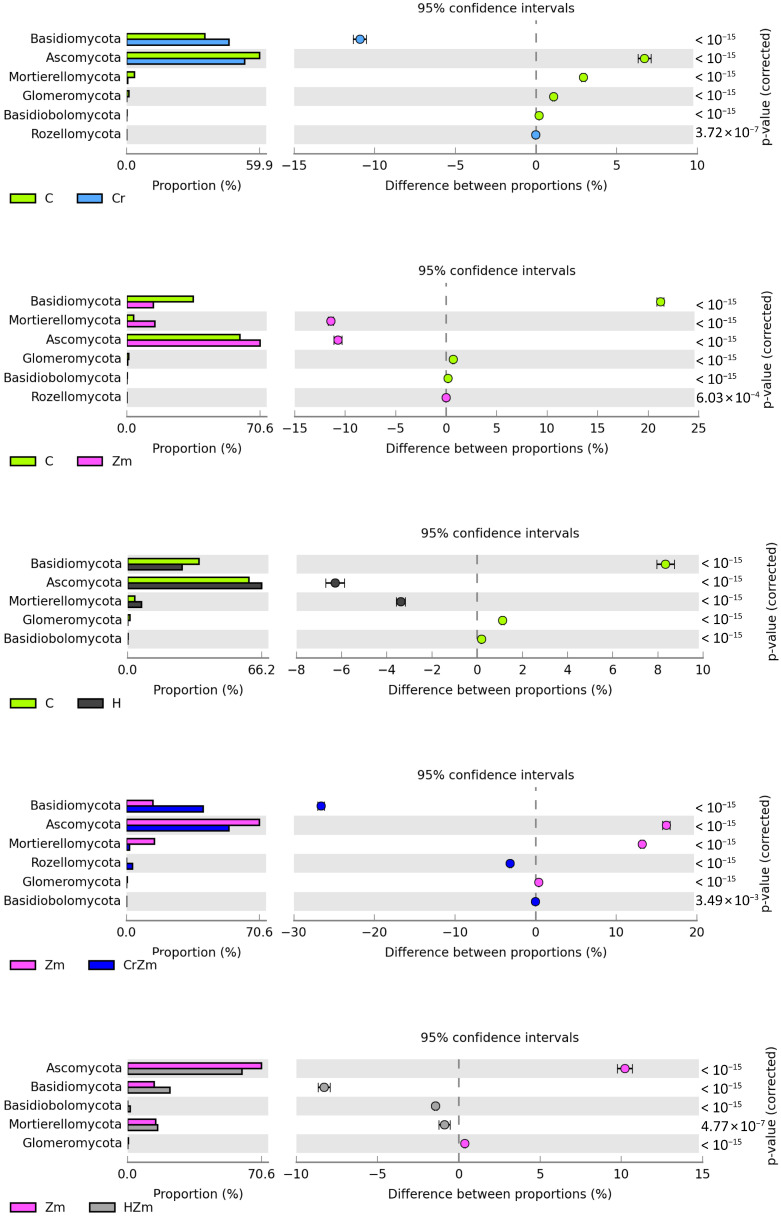
Effect of Cr (VI), *Zea mays* and HumiAgra on the relative abundance of dominant fungi phylum. C—unpolluted unsown soil; Cr—soil polluted with chromium (VI); H—soil with the addition of HumiAgra; Zm—soil sown with *Zea mays*.

The analyzed soil, regardless of the tested variables, was dominated by fungi of the phyla *Ascomycota*, *Basidiomycota* and *Mortierellomycota* (Figure 7), whose average per cent share in the structure, irrespective of an experimental variant, was 63.35%, 29.34% and 6.39%, respectively. The contamination of bare soil with Cr(VI) contributed to a decrease in the abundance of *Ascomycota* by 6.73% and *Mortierellomycota* by 2.93% while inducing a rise in the number of *Basidiomycota* bacteria by 10.92%. The effect of HumiAgra was contrary to the above, as the substance added to soil increased the share of *Ascomycota* fungi by 6.28% and *Mortierellomycota* fungi by 3.38% while lowering the percentage of *Basidiomycota* fungi by 8.35%.

The response of fungi to the sowing of soil with *Zea mays* was similar to that caused by HumiAgra, as the cultivation of maize resulted in an increase in the count of *Ascomycota* by 10.69% and *Mortierellomycota* by 11.41%, while the percentage of *Basidiomycota* decreased by 21.22%. The response of the fungal phyla to the pollution of soil under maize with Cr(VI) was analogous to what was observed in Cr(VI) polluted bare soil. The former resulted in a decrease in the abundance of the phylum *Ascomycota* by 16.21% and *Mortierellomycota* by 13.21%, while raising the share of *Basidiomycota* by 26.63%. When soil fertilized with HumiAgra was seeded with *Zea mays*, the abundance of the phylum *Mortierellomycota* in the total structure of fungi decreased by 8.92% and that of *Basidiomycota* was lowered by 4.59%.

**Figure 8:**
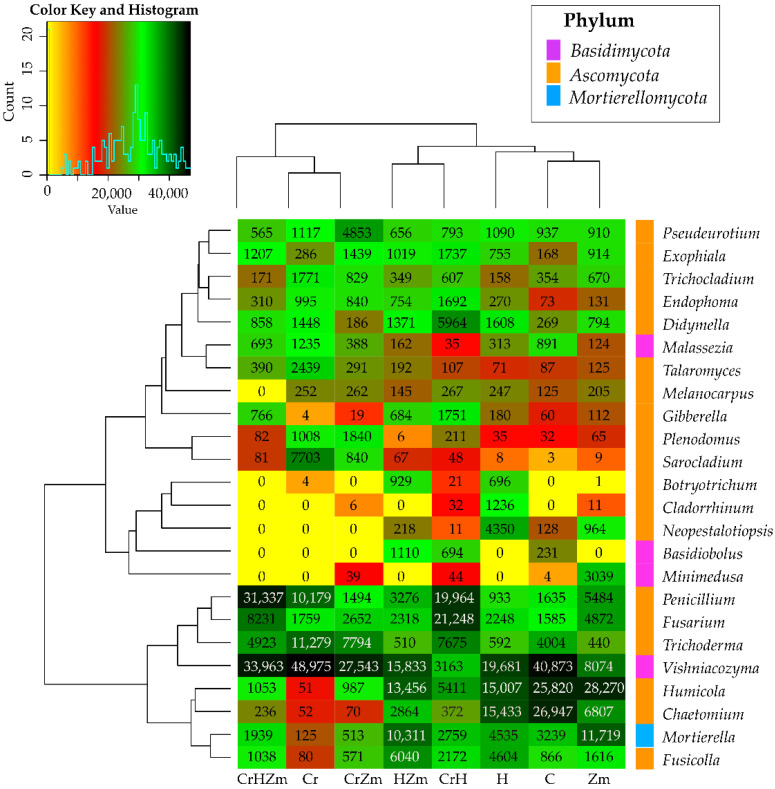
Effect of Cr(VI), *Zea mays* and HumiAgra on the relative abundance of dominant fungal genus. C—unpolluted unsown soil; Cr—soil polluted with chromium (VI); H—soil with the addition of HumiAgra; Zm—soil sown with *Zea mays*.

Nineteen genera of fungi were identified within the phylum *Ascomycota*, four from the phylum *Basidiomycota* and one from the phylum *Mortierellomycota* (Figure 8).

The phylum *Ascomycota* was dominated by *Humicola* (on average 11,257 OTU), *Penicillium* (9288 OTU), *Chaetomium* (6598 OTU), *Fusarium* (5614 OTU) and *Trichoderma* (4652 OTU). The phylum *Basidiomycota* was dominated by *Vishniacozyma* (24,763 OTU), and the phylum *Mortierellomycota* by *Mortierella* (4393 OTU). The OTU number of the remaining genera varied from 161 (*Cladorrhinum*) to 2123 (*Fusicolla*). In the soil not cropped with *Zea mays* and not contaminated with Cr(VI), the most numerous fungus belonged to the genus *Vishniacozyma* (40,873 OTU) from the phylum *Basidiomycota*, followed by the phylum *Ascomycota* and the genera *Chaetomium* (26,947 OTU), *Humicola* (25,820 OTU) and *Trichoderma* (4004 OTU), as well as *Mortierella* (3239 OTU) from the phylum *Mortierellamycota*. Other quite numerous fungi were *Penicillium* (1635 OTU) and *Fusarium* (1585 OTU) from the phylum *Ascomycota*. The pollution of bare soil with Cr(VI) induced significant changes in the abundance of fungal genera.

**Figure 9:**
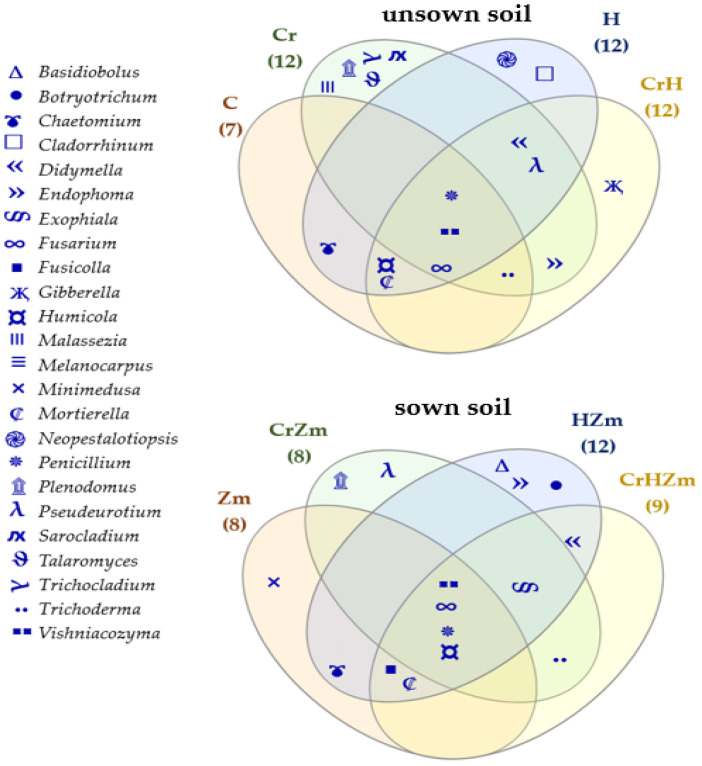
Venn diagram showing unique and common types of fungi in unsown and sown *Zea mays* soil. C—unpolluted unsown soil; Cr—soil polluted with Cr (VI); H—soil with the addition of HumiAgra, CrZm—soil sown with *Zea Mays* contaminated with Cr (VI); HZm—soil sown with *Zea Mays* with HumiAgra.

Under the influence of this element, the OTU of *Trichoderma* rose from 4004 to 11,279, *Penicillium* from 1635 to 10,179, *Sarocladium* from 3 to 7703, *Tallaromyces* from 87 to 2439, *Trichocladium* from 354 to 1771, *Didmella* from 269 to 1448, *Malassezia* from 891 to 1235, *Plenodomus* from 32 to 1008 and *Endophoma* from 73 to 995, while the OTU of *Chaetomium* and *Humicola* decreased drastically from 26,947 to 52 and from 25,820 to 51, respectively. When soil was sown with *Zea mays*, an increase was observed in the OTU of fungi from the genera *Minimedusa*, *Mortierella*, *Penicillium*, *Fusarium*, *Fusicolla*, *Neopestalotiopsis* and *Exophiala*, while the OTU of *Trichoderma, Vishniacozyma*, *Malassezia* and *Chaetomium* decreased. A positive effect of chromium on some genera of fungi, e.g., *Vishniacozyma*, *Pseudeurotium*, *Trichoderma* and *Plenodomus*, was observed in the soil under *Zea mays* polluted with Cr(VI). Its negative influence was detected in the same experimental variant on other fungal genera, such as *Humicola*, *Mortierella*, *Chaetomium* and *Minimedusa*. The direction of the effect produced by HumiAgra on genera of fungi in bare soil and in soil under maize was not always the same. Namely, a very positive effect of this fertilizer was observed in both soils on the development of *Botryotrichum*, *Didymella, Exophiala* and *Endophoma*. Moreover, in the soil not cropped with maize, HumiAgra stimulated the development of *Cladorrhinum*, *Neopestalotiopsis* and *Fusarium*, whereas in soil under maize, it positively affected the development of *Vishniacozyma* and *Fusicolla*. Furthermore, this preparation, irrespective of the soil use, decreased the OTU of *Chaetomium* and *Humicola* and caused the same effect on the genera *Vishniacozyma* and *Trichoderma* in the soil not sown with *Zea mays*. The Venn diagram shown in Figure 9 indicates that the core of the soil mycobiome in soils not sown with *Zea mays* and under this plant, regardless of the Cr(VI) pollution or the application of HumiAgra, was composed of fungi of the genera *Vishniacozyma*, *Penicillium* and *Fusarium.*

The unique mycobiome in soils exposed to Cr(VI) was noteworthy; in unsown soils, mycobiome was represented by fungi of the genus: *Malassezia, Talaromyces*, *Trichocladium*, *Plenodomus* and *Sarocladium*, whereas only one of these genera, namely *Plenodomus*, was also detected in the mycobiome of soils not cropped with *Zea mays*.

**Figure 10:**
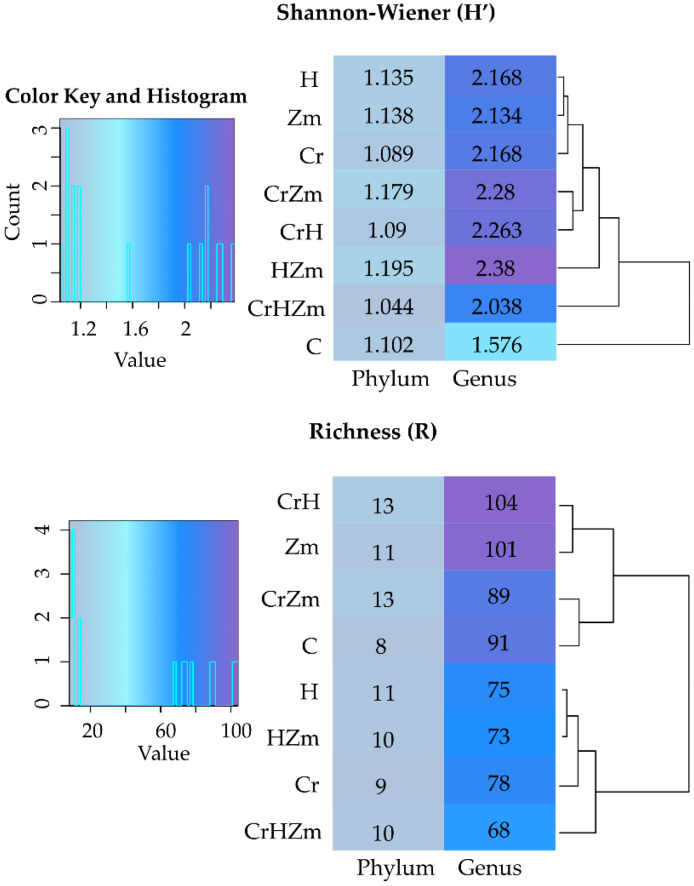
Shannon-Wiener index (H’) and the richness (R) of fungi in the soil. C—unpolluted unsown soil; Cr—soil polluted with Cr (VI); H—soil with the addition of HumiAgra; Zm—soil sown with *Zea mays*.

The values of the Shannon-Wiener diversity (H’) and richness (R) indices demonstrated that Cr(VI) only very slightly modified the diversity of fungi (Figure 10), and these modifications only concerned genera. Under the impact of this element in the bare soil, the Shannon-Wiener diversity index was higher, while the richness index decreased. The values of both indices increased for phyla and genera in response to maize cultivation. On the other hand, Cr(VI) and HumiAgra in bare soil increased the diversity of fungi but reduced the richness of genera.

## 3. Discussion

### 3.1. Zea mays Reaction to Soil Contamination with Cr(VI)

No convincing evidence has been obtained so far to confirm that chromium is an essential element for plants, including maize. Studies carried out over the past thirty years unquestionably emphasize the toxic effect of chromate oxyanion Cr(VI) on the growth and development of plants as a result of oxidative stress [54]. Thus, it could be predicted that the pressure of Cr(VI) applied to soil would be reflected in a decrease in the yield of both the aerial parts and roots of maize. These observations correspond to the findings of Mohammed et al. [55], who reported that oxyanion decreased the germination rate, root biomass and dry matter of shoots. In an experiment conducted by Polti [56], plants growing in the presence of Cr(VI) demonstrated macroscopic damage, such as 25%, 73% and 80% shorter lengths of roots, stems and leaves, respectively, relative to control. Among other key physico-biochemical responses linked to the phytotoxicity of Cr ions, the chlorosis of leaves, necrosis, withering, retarded development of lateral roots and the desynchronization of the antioxidant defense system resulting in the disruption of the morphophysiological processes of cultivated plants are worth mentioning [57,58]. To some extent, this is a consequence of delayed germination of seeds due to the inhibited activity of α and β-amylase [59], responsible for the hydrolysis of starch, which induces this stage in the development of maize [58]. It is also important to mention that the value of the *Zea mays* greenness index decreases due to the damage to the plant’s photosynthetic apparatus and resultant inhibition of the synthesis of chlorophyll a and b [60], as well as some damage in the structure of chloroplasts [61]. According to Rodriguez et al. [62] and Sharma et al. [39], exposure to Cr(VI) induces the overproduction of reactive oxygen species (ROS) which inhibits the expression of genes engaged in photorespiratory pathways and disturbs the activity of δ-aminovolulic acid dehydratase, which is significant in the synthesis of chlorophyll. Inhibition of photosynthesis is also caused by the binding of Cr ions with the cytochrome hem group, blocking the transport of electrons by changing the Fe and Cu redox state [63]. Higher bioaccumulation of chromium in roots (BF_R_) than in aerial organs of plants (BF_A_) arises from the fact that a first structure, i.e., the hypocotyl, is the first target of the toxicity of Cr(VI), which accumulates preferentially in the root tip, inhibiting mitosis and elongation of cells [58]. It is worth underlining that the growth of the primary root and adventitious roots, i.e., those produced in response to stress conditions, is controlled by the Solitary Root transcription factor [54]. However, this factor does not protect the root against the toxic effect of Cr(VI), which, as reported by Singh et al. [64], adversely affects the formation of capillaries and the division of root cells, while the excessive generation of ROS leads to the peroxidation of membrane lipids. Toxicity of Cr(VI) is correlated with the accumulation of H_2_O_2_ in maize roots [32]. The lower mobility of Cr(VI) is noteworthy, as it results in the accumulation of this metal in plant roots. Meanwhile, Cr(III) is transported from roots to shoots and other aerial parts of plants. The mechanism is based on the reduction of Cr(VI) to Cr(III) induced by the apoplast system during transport to xylem. As a consequence, excess amounts of Cr(III) are precipitated in the cell and eventually undergo complexation with ligands, such as carboxylates, as a result of which small concentrations of Cr may be found in the aerial organs of plants [65]. Unfortunately, this does not protect the plant from the effects of Cr exposure caused by excessive production of ROS, which include disruption of chromosome aberrations, DNA strand breaks, or inhibition of cell division, eventually leading to cell death [58]. In a study by Terzi and Yıldız [32], 46 proteins with varied expression were identified. Among them, 41.3%, including peroxyredoxine-5 (Prx%), glutathione peroxidase (GTS), superoxide dismutase [Mn] (MSD) and S-glutathione transferase (GTS), participated in the biological pathways responsible for plant stress response. Plants are also protected from oxidative damage by auxin, ethylene and jasmonic acid [54]. According to Mohammed et al. [55], higher Cr toxicity also induces an increase in the amounts of proline and phenols. 

A significant role in preventing Cr(VI) retention is also using amino acids and organic acids [66]. Hence, the application of HumiAgra resulted in higher values of the translocation factor (TF) achieved by maize and alleviated the inhibitory effect of Cr(VI) on plant growth and development. This effect is explained by the formation of humic acid chelates with metal ions, which decrease the bioavailability of this pool of xenobiotics, an effect which carboxylic groups, inter alia, are responsible for [67,68].

### 3.2. Reaction of Bacteria and Fungi to Soil Contamination with Cr(VI)

#### 3.2.1. Breeding Microorganisms

The resistance of microorganisms to Cr(VI) is an intricate issue. It is associated with the biological availability of xenobiotics, as well as the environmental conditions that significantly moderate Cr(VI) toxicity. These include temperature, pH and the availability of electron donors, such as lactate, glucose, sucrose, formate or NADH [69,70]. However, considering the generally accepted paradigm of Cr(VI) toxicity, it was to be expected that this metal ion would have an inhibitory effect on the groups of microorganisms analyzed in this experiment. It is known that chromium compounds cause genotoxic effects, including chromosomal aberration and the transformation of cells [71]. Cr(VI) is highly soluble and mobile in the environment [72], and its toxic effect is linked to the high oxidative potential of chromates [CrO_4_^2−^], which leads to the generation of free radicals during the reduction of Cr(VI) to Cr(III) [73]. Thus, a negative effect of Cr(VI) on counts of organotrophic bacteria, actinomycetes and, to a lesser extent, fungi was noted in our research. In view of the fact that Cr(III), as the most thermodynamically stable form of chromium in soil, precipitated to chromium hydroxide [Cr(OH)_3_], considered to be less toxic [74,75], the response of microorganisms might not have been too obvious. In a study by Wyszkowska et al. [76], it was demonstrated that Cr(VI) significantly inhibited actinomycetes and Azotobacter sp. and moderately inhibited the population of fungi. Nevertheless, it stimulated the proliferation of oligotrophic, copiotrophic, ammonifying and nitrogen immobilizing bacteria. Several enzymes inducing respiratory process that can reduce Cr(VI) to Cr (III) were identified in these microorganisms; these include intracellular Cr(VI) reductases, such as AzoR, Frp, YcnD, NfoR, ChrR, YieF, FerB, NfsA and NfsB, NemA and CsrF [77]. However, Wang and Cui [78] reported that absorption of Cr(III) on the surface of a bacterial cell may result in a change in the cell’s protein composition, manifested by topological protrusions after the interaction of chromium with bacteria. According to Ramirez-Dias et al. [79], Cr(III) may also be responsible for mutagenesis and changes in the structure and activity of enzymes resulting from their reaction with other carboxyl or thiol groups. To identify the causes of the adverse impact of Cr(VI) on the counts of the microorganisms detected in this study, we should also take into account the fact that the biosorption of this element on the surface of bacterial cells is a physicochemical process, while bioreduction with the participation of cytosolic molecules is a purely chemical process, and its kinetics increase with increasing temperatures [70]. This tendency may have contributed to the elimination of mesophilic microorganisms by intensifying the efficiency of thermophilic bacteria [80]. The elevation of temperature might have also caused inactivation of Cr(VI) reductase [81]. In an analysis of the present study results, it is also appropriate to refer to the importance of pH as a factor moderating the biosorption of Cr(VI) on the surface of bacterial cells. Lower pH intensifies the biosorption of chromium present as HCrO^4−^ and CrO_2_^7−^ through an increase in the protonation of carboxyl and amine groups. In turn, an increase in pH due to the deprotonation of functional groups, and therefore, electrostatic repulsion between negatively charged Cr(III) and Cr(VI) and the surface of a cell, slows down this process [82,83]. The importance of this parameter extends to the functions of humic acids, which are strongly dependent on pH. Humic acids, as reservoirs of electron donors/acceptors, also contribute to the reduction of inorganic pollutants, including Cr(VI) [84,85,86], which, in our study, translated into the alleviation of the inhibitory effect of this element on breeding microorganisms following the application of HumiAgra. In a study by Zaborowska et al. [87], humic acid was also found to stimulate the multiplication of organotrophic bacteria.

The negative response of actinomycetes to the application of Cr(VI) to soil was a controversial finding. Considering the results of a study completed by Polti et al. [88], in which a strain Streptomyces sp. MC1 was shown to be able to reduce the bioavailability of Cr(VI) by 90% in soil polluted with 50 mg Cr kg^−1^ d.m. of soil, and the report of Laxman and More [89] regarding the reducing potential of Streptomyces griseus (therefore participating in the conversion of Cr(VI) to Cr(III)), one would expect stimulated proliferation of this group of microorganisms. Yet, this was only observed in soil cropped with *Zea mays*. This dependence was confirmed by the observations reported by Polti et al. [90], who documented both the stimulation of the growth of roots and biomass of *Zea mays* by Streptomyces sp. MC1, as well as decreased accumulation of this element in the plant.

The fact that exposure to Cr(VI) in our study enhanced the development of strategy K fungi can be explained by the ability of this group of microorganisms to adsorb inorganic pollutants on the surface of the cell wall in a way corresponding to its composition [91]. Positively charged groups, including amine, sulfhydryl, carboxyl and hydroxyl ones, are responsible for the binding of metals [92]. According to Canton et al. [93], the mechanism of detoxication of fungi, beside adsorption and Cr(VI) reduction, also involves cytoplasmic chelation, subcellular deisolation and repair of damaged biomolecules.

#### 3.2.2. Bacteria and Fungi Identified by the NGS Method

*Actinobacteria* and *Acidobacteria*, as well as *Proteobacteria*, are the most frequently mentioned representatives of the soil bacterial phyla [94,95,96]. *Actinobacteria* and *Proteobacteria* were the dominant phyla in the analyzed soil, although Cr(VI) moderated the structure of bacteria, decreasing the number of the Proteobacteria cells while stimulating an increase in the abundance of *Actinobacteria* in bare soil. In the soil sown with *Zea mays*, Cr(VI) induced an increase in the abundance of OTU of *Proteobacteria*. The results of this research show that the soil treatment with 60 mg Cr(VI) kg^−1^ d.m. of soil promoted the multiplication of bacteria, including the genera *Terracoccus*, *Agrobacterium* of the phylum *Proteobacteria*, and the genus *Arthrobacter* representing the phylum *Actinobacteria*. The observed tendencies may be attributed to the bioremediation potential of the bacteria assigned to these taxa, which are distinguished by the highest diversity and abundance of genes resistant to heavy metals, including Cr(VI) [97]. This bioremediation potential also relies on several mechanisms of tolerance to this element, including the absorption and effect of Cr(VI), detoxication of free radicals (ROS) and DNA repair systems [98]. The inhibitory effect of Cr(VI) on *Pseudomonas* and *Cellulosimicrobium* in bare soil was surprising, because the dominance of these genera in soil polluted with Cr(VI) and sown with *Zea mays* was observed. According to Karthik et al. [99], in response to the toxicity of this element, *Cellulosimicrobium funkei* AR8 employs both extracellular and intracellular reduction. Its bioreduction potential toward Cr(VI) was also confirmed by Bharagava and Mishra [100]. Numerous studies have shown that bacteria of the genus Pseudomonas have a high capacity for adsorption, bioreduction and influence on Cr(VI) [101,102,103]. The effective conversion of Cr(VI) to Cr(III) catalyzes several reductases, which *Pseudomonas* bacteria are equipped with. These include flavin reductases, NAD(P)H-dependent reductases or ferric reductase [104]. It is also worth underlining that an increased efflux of Cr(VI) in *Pseudomonas aeruginosa* is linked to the overexpression of the CHrA efflux protein, located on plasmids or chromosomes, and of the ChrB and ChrE proteins [105,106]. Whereas it is true that deletion of the ChrA gene inactivates the efflux system [107], the highest metabolic capability of Cr(VI) was demonstrated in the case of the co-expressed chRAB gene [108]. The efflux mechanism can be distorted by sulfate or molybdate—due to the fact that their structures are similar to that of chromate—and the resultant competition for the ChrA protein [109]. A significant mechanism of resistance of *Arthrobacter* and *Agrobacterium* to Cr(VI) is the production of exopolysaccharide (EPS) [110,111], which forms an extracellular complex with Cr(III), owing to the negatively charged carboxyl and hydroxyl groups [112]. In turn, the positive response of *Terracoccus* to the soil application of Cr(VI) can be attributed to the high content of anion polymers in the cell wall of Gram-positive bacteria and the affinity between ligands of the cell surface, such as phosphoryl, SO_3_^2−^, RNH_2_ or R_2_NH, and chromium cations [113]. The high biosorption potential of Cr(VI) by the Gram-negative bacteria identified in this study is most probably a consequence of the increased release of negatively charged lipopolysaccharides that facilitate the binding of chromium on the cell surface [114]. Sowing the soil with *Zea mays* stimulated the abundance of OTU of the vast majority of bacteria. Endocrine chelates in the plant roots are responsible for the formation of insoluble compounds which bind Cr(VI) with vacuoles and the cell wall. This process reduces the toxicity of chromium toward bacteria [64]. According to Wang et al. [115], fibrinolytic enzymes, *β*-1,4-xylosidase (BX) and cellobiohydrolase (CBH) are significant coadjutants alleviating the negative impact of Cr(VI) on rhizosphere bacteria. Their activity, however, is correlated with the content of soil organic matter (SOM). SOM comprises humic acids, which, owing to the presence of carboxyl, carbonyl, phenolic, hydroxyl and quinone functional groups joined by carbon chains, aspire to be effective chromium sorbents.

Exposure to Cr(VI) has an effect on the biodiversity of fungi. Regardless of the type of soil used, at the phylum level, an escalation of *Basidiomycota* OTU abundance and a decrease in *Ascomycota* and *Mortierellomycota* OTU values were noted. In turn, at the taxonomic level of genera, soil contamination with chromium led to the highest share of *Trichoderma* and *Penicillium*. These results were confirmed by the study of Benila Smily and Sumithra, [116], where *Trichoderma* sp. BSCRO2 demonstrated the ability of Cr(VI) biosorption, and the observations of Saranya et al. [117], revealing the reducing potential of *Trichoderma asperellum*. Cr(VI) binding sites on the surface of *Trichoderma* sp. cells were determined to be carboxyl and amine groups [118]. Arevalo et al. [119] also demonstrated the bioregeneration potential of *Penicillium* sp., effectively reducing Cr(VI) to Cr(III). Like with bacteria, *Penicillium janthinellum* P1 was determined to contain the chrA gene, encoding the ChRA protein. This protein acts as a chemoosmotic pump, inducing a strategy of chromate efflux from the cytoplasm or periplasm into the extracellular matrix [120]. It should also be mentioned that the tolerance of Penicillium is a consequence of the activation of cellular transporters eliminating Cr(VI) from the cell membrane of fungi. Apart from the ChRA protein, these include SEC23 and SEC24 transport proteins. The positive response of the genera of fungi identified in our study can also be attributed to the mechanism of the removal of Cr(VI) from the plasma membrane by cells chelating the sequestration of this element, i.e., metallothioneins [121].

## 4. Materials and Methods

### 4.1. Soil Characteristics

The soil used in this pot experiment was taken from the topsoil (0–0.20 m) of an agriculturally used field, situated in the northern part of the Olsztyn Lake District, which lies on the East European Plain (NE Poland, 53,72° N, 20,42° E). According to the grain-size classification of the International Union of Soil Sciences and the United States Department of Agriculture [122], the soil represented sandy loam. The soil sampled was transported to a university greenhouse, passed through a 0.5 mesh sieve, and used to start pot experiments. In parallel, some of the soil was sifted through a 2 mm mesh sieve and submitted to basic physicochemical analyses. The characteristics of the soil and a basic description of the design of the experiment are shown in Table 4.

### 4.2. Study Design

Pot experiments were carried out in a greenhouse. First, 3.5 kg batches of sandy loam were placed in polyethylene pots. *Zea mays*, a crop which is cultivated worldwide [123], was selected as a phytoremediation plant. Maize is now one of the two most popular crops in the world [124]. AL-Huqail and El-Bondkly [125] and Meers et al. [126] draw attention to its usefulness for the phytoremediation of heavy metals from polluted soils. The biomass thus harvested could be used for alternative energy generation [127,128]. In order to gain a better understanding of the effect of Cr(VI) on the soil microbiome, the whole experiment was divided into two series: bare soil and soil sown with *Zea mays*. The following experimental variants were included in each series: (1) unpolluted soil, (2) soil polluted with Cr(VI), (3) soil fertilized with HumiAgra and (4) soil polluted with Cr(VI) and fertilized with HumiAgra. In all experimental variants, an identical N, P, K and Mg fertilization regime was maintained. Soil in variants 2 and 4 was contaminated with K_2_Cr_2_O_7_ in an amount of 60 mg Cr(VI) kg^−1^ d.m. of soil. Chromium in the form of Cr(VI) was chosen for the study, as it is one of the most toxic metals in the environment [129,130]. Cr(VI) is one of the highest priority pollutants [129,130]. In order to limit the negative effect of Cr(VI) on the yields of *Zea mays* and to biostimulate the natural soil microbiota, humic acids in the form of the preparation HumiAgra (AgraPlant, Kielce, Poland) were applied to soil. Mineral fertilizers were applied once, prior to placing the soil in pots. In the four-leaf stage (BBCH 14) and the nine-leaf stage (BBCH 19), the Soil and Plant Analysis Development (SPAD) leaf greenness index was determined in eight replicates. The measurements were made with a SPAD 502 Chlorophyll Meter 2900P (KONICA MINOLTA, Inc. Chiyoda, Japan). In the stage BBCH 51 (beginning of tassel emergence), the yields of aerial parts and roots of maize were determined. At harvest, the height of the plants was measured, and the yield of aerial parts and roots was determined. The plants were dried at 60 °C for four days.

### 4.3. Methods of Soil Microbiological Analyses

Breeding microorganisms. Counts of organotrophic bacteria [131], actinomycetes [132] and fungi [133] were determined in four replicates in fresh, moist soil passed through a 2 mm mesh sieve, using the serial dilution method. The culture conditions and the exact procedure for the isolation of microorganisms were described in our earlier paper [134]. Colony forming units (cfu) of microorganisms are presented per 1 kg d.m. of soil.

Bacteria and fungi identified with the NGS method. The isolation of DNA from soil was performed with a Genomic Mini AX Bacteria+” kit (A&A Biotechnology). Universal starters 1055F (5′-ATGGCTGTCGTCAGCT-3′) and 1392R (5′-ACGGGCGGTGTGTAC-3′) amplifying a fragment of the bacterial gene 16S rRNA and ITS were used in the reaction [135]. The sequencing of the Bacteria and Archaea amplicons was conducted on the basis of the hypervariable region V3-V4 of the gene 16S rRNA, while that of fungal amplicons was based on the ITS1 region. Specific sequences of primers 341F (5′-CCTACGGGNGGCWGCAG-3′) and 785R (5′-GACTACHVGGGTATCTAATCC-3′) served to amplify bacteria, while ITS1FI2 (5′-GAACCWGCGGARGGATCA-3′) and 5.8S (5′-CGCTGCGTTCTTCATCG-3′) were used for the amplification of fungi.

Detailed PCR conditions were described in our previous articles [87,136]. The sequencing was done in the company Genomed SA (Warsaw, Poland). In order to classify the readings of bacteria to the level of particular taxonomic classes, a bioinformatics analysis was run in the Quantitative Insights Into Microbial Ecology (QIIME) software package, according to the GreenGenes reference sequences database (version 13.8), and UNITE for the classification of fungi. The article presents results of relative abundances of the identified sequences at the two taxonomic levels (phylum and genus) where OTU exceeded 1%. Data on the sequencing of bacteria and fungi were deposited in the GenBank NCBI (https://www.ncbi.nlm.nih.gov/under access numbers: Prokaryotic 16S rRNA https://www.ncbi.nlm.nih.gov/nuccore/?term=OP832646:OP834010[accn] (accessed on 20 November 2022), Eukaryotic Nuclear rRNA/ITS: https://www.ncbi.nlm.nih.gov/nuccore/?term=OP798782:OP799344[accn] (accessed on 13 November 2022).

### 4.4. Physicochemical and Chemical Analyses of Soil

Prior to the experiment, the grain-size distribution of the soil was assessed with the aerometric method, and the physicochemical and chemical properties of the soil were determined. All determinations were made based on three replicates. The physicochemical analyses included: determination of pH in 1 mol KCl dm^−3^, as well as hydrolytic acidity (HAC) and sum of exchangeable base cations (EBC). Cation exchange capacity (CEC) and alkaline cation saturation (ACS) were computed from the values of HAC and EBC. Other determinations were: content of total nitrogen (N_tot_), organic carbon (C_org_), assimilable P, K and Mg, and exchangeable cations, i.e., Ca^2+^, Mg^2+^, K^+^ and Na^+^. All these determinations were made with standard methods, as described in our earlier papers [87,137]. In addition, both before the experiment and after harvest, the concentrations of chromium in soil and in the aerial parts and roots of *Zea mays* were determined with flame atomic absorption spectrometry [138,139] using an Agilent Technologies, type: AA-280 (Agilent Technologies, Santa Clara, CA, USA) atomic absorption spectrophotometer. Samples for the determination of the chromium content were mineralized in aqua regia.

### 4.5. Data Analysis and Statistical Elaboration

By taking into account the yield of *Zea mays* aerial organs and roots, as well as the content of chromium in soil and plant, the uptake of chromium by maize (D) and parameters evaluating the usefulness of *Zea mays* for remediation of Cr(VI) polluted soil, such as tolerance index (TI), translocation index (TI), bioaccumulation in aerial parts (BF_A_), bioaccumulation in roots (BF_R_) and accumulation factors (BF) were calculated. For this purpose, the formulas described in our previous articles were used [6,50]. Moreover, using the formulas proposed by Wyszkowska et al. [136], the influence of Cr(VI) and humic acids on breeding microorganisms was assessed. By counting colony forming units of these microorganisms daily for ten days, we were able to determine the colony development index (CD) and ecophysiological diversity index (EP) [140] of bacteria, actinomycetes and fungi. By taking the determined values of OTU of bacteria and fungi at the levels of phylum and genus, values of the following indicators related to diversity of microorganisms were calculated: the Shannon-Wiener (H’) and the richness (R) indices [141].

All results were processed statistically, with the help of a Statistica 13.3 package [142] R v1.2.5033 software [143] with supplementary R v3.6.2 [144] and the gplots library [145]. In order to determine the impact of Cr(VI) and humic acids on the yield and height of plants, the SPAD greenness index, the number of cultured microorganisms, the CD and EP index, an analysis of variance (ANOVA) was performed at the significance level of *p* = 0.05. Homogenous groups were counted with the help of the Tukey’s post-hoc test. Of all microorganisms identified with the NGS method, the ones with OTU ≥ 1% were selected for further analysis. The gathered metagenomic data of the bacterial and fungal phyla were compared statistically using the G-test (w/Yates’) + Fisher’s, with the help of STAMP 2.1.3. software [146]. In turn, the genera of the microorganisms were visualized on heat maps using the R application, with a dendrogram showing their similarities [143,144,145]. The composition of the key assemblages of bacteria and fungi, composing the core microbiome defining the unique characteristics of unsown soils and soils sown with *Zea mays*, was presented using InteractiVenn [147].

## 5. Conclusions

Soil contamination with Cr(VI) at a dose of 60 mg kg^−1^ d.m. significantly inhibited the growth and development of *Zea mays*, which, as a consequence of this pollution, scored lower on the leaf greenness index and yield mass parameter. The quality of maize also deteriorated due to the elevated accumulation of chromium in both aerial organs and roots. Chromium also decreased the abundance of breeding microorganisms and distorted the proportions between strategy r and strategy K microorganisms, as well as the ecophysiological diversity of fungi. The adverse influence of Cr(VI) on *Zea mays* and on breeding microorganisms was significantly reduced by the application of HumiAgra to soil contaminated with this metal. Fungi were more tolerant to the effect of Cr(VI) than organotrophic bacteria and actinomycetes. Chromium (VI) also effected changes in the structure of assemblages of bacteria and fungi, as determined using the NGS method. The metal modified the genetic diversity of bacteria more than that of fungi. This element had an unambiguously negative effect on bacteria *Cellulosimicrobium*, *Kaistobacter*, *Rhodanobacter, Rhodoplanes* and *Nocardioides* and fungi *Chaetomium* and *Humicola*. In contrast, it promoted the development of bacteria of the genera *Terracoccus, Paenibacillus, Phyciococcus, Agrobacterium, Arthrobacter, Kribbela, Devosia, Burkholderia* and *Ramibacter* and fungi *Trichoderma, Penicillium, Sarocladium, Tallaromyces, Trichocladium, Didmella, Malassezia, Plenodomus* and *Endophoma*. The adverse effect of Cr(VI) on the diversity of bacteria and fungi was moderated by the cultivation of *Zea mays* and the application of HumiAgra. The core microbiome shared by all the experimental objects was composed of bacteria of the genera *Kaistobacter, Terracoccus and Cellulosimicrobium* and fungi of the genera *Vishniacozyma, Penicillium* and *Fusarium*. The research results suggest that *Zea mays* and HumiAgra can be helpful for the amelioration of soil contaminated with Cr(VI).

## Figures and Tables

**Table 1:** Yield of *Zea mays* (g d.w. pot^−1^), greenness index (SPAD) and plant height (cm).

Dose of Cr(VI), mg kg^−1^ d.m. of Soil	Yield, (d.m. g Pot^−1^)		SPAD BBCH *Zea mays*		Plant Height, cm
	Aerial Parts	Roots	14	19	
Control					
0	44.923 ^b^	16.324 ^a^	32.394 ^a^	27.129 ^b^	138.800 ^b^
60	4.363 ^c^	1.359 ^c^	17.156 ^c^	19.446 ^c^	70.500 ^c^
HumiAgra					
0	52.567 ^a^	15.242 ^ab^	32.598 ^a^	27.838 ^b^	162.818 ^a^
60	49.624 ^a^	14.088 ^b^	32.481 ^a^	33.136 ^a^	146.750 ^b^

Homogeneous groups according to yield, SPAD and plant height are marked with identical letters (a–c).

**Table 2:** Chromium content, mg kg^−1^ d.m.

Dose of Cr(VI), mg kg^−1^ d.m. of Soil	Soil Use		Aerial Parts	Roots
	Unsown	Sown with *Zea mays*		
Control				
0	12.375 ^d^	15.075 ^d^	0.830 ^c^	3.170 ^c^
60	69.825 ^b^	72.525 ^b^	1.040 ^a^	17.670 ^a^
HumiAgra				
0	19.500 ^c^	18.900 ^c^	0.660 ^d^	1.230 ^d^
60	76.950 ^a^	76.350 ^a^	0.950 ^b^	8.010 ^b^

Homogeneous groups according to chromium content in soil, aerial parts and roots are marked with identical letters (a–d).

**Table 3:** Chromium uptake by *Zea mays* (D), tolerance index (TI) and chromium translocation factors (TF), aerial bioaccumulation (BF_A_), root bioaccumulation (BF_R_) and accumulation (BF).

Dose of Cr(VI), mg kg^−1^ d.m. of Soil	D µg pot^−1^	TI	TF	BF_A_	BF_R_	BF
	Control					
0	89.033 ^b^	-	0.262 ^b^	0.055 ^a^	0.210 ^b^	0.265 ^a^
60	28.549 ^d^	0.093	0.059 ^d^	0.014 ^c^	0.244 ^a^	0.258 ^a^
	HumiAgra					
0	53.441 ^c^	-	0.537 ^a^	0.035 ^b^	0.065 ^d^	0.100 ^c^
60	159.989 ^a^	0.940	0.119 ^c^	0.012 ^c^	0.105 ^c^	0.117 ^b^

Homogeneous groups according to uptake and each index are marked with identical letters (a–d).

**Table 4:** Design of the greenhouse experiments with *Zea mays*.

Location	University of Warmia and Mazury in Olsztyn, Poland		
An experimental plant	*Zea mays* L. variety LG 32.58 (variety registered in European Union), eight seeds were sown in a pot; after emergence, four plants were left in the pot		
Soil	Sandy loam: clay < 0.002 mm–3.71%, silt 0.02–0.05 mm–32.68% and sand–0.0–2.0 mm–63.61%.		
	Physicochemical properties:		
	pH_KCl_–4.40 HAC–26.10 EBC–63.60 CEC–89.70	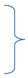	mmol (+) kg^−1^ d.m.
	ACS–70.90%		
	Exchangeable cations:		
	K^+^ 168.00 Ca^2+^ 1190.50 Na^+^ 10.00 Mg^2+^ 82.10	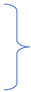	mg kg^−1^ d.m.
	Chemical properties per 1 kg d.m: N_tot_ 0.83 g, C_org_ 10.00 g, P_av_ 81.10 mg, K_av_ 145.25 mg, Mg_av_ 71.00 mg, Cr_tot_ 12.37 mg		
Mineral fertilisation	mg kg^−1^ of d.m. soil: N 140 [CO(NH_2_)_2_], P 50 [KH_2_PO_4_], K 140 [ KH_2_PO_4_ + KCl], Mg 20 [MgSO_4_ × 7H_2_O] The form of fertilizer is given in parentheses		
Soil contamination with Cr(VI)	60 mg Cr(VI) kg^−1^ d.m. of soil in form of K_2_Cr_2_O_7_		
Use of biostimulation	HumiAgra (AgraPlant, Kielce, Poland) is an ecological product, contains 90% humic acids (50% humic acids and 50% fulvic acids). Dark brown powder, pH 8–10. Contains 8% K_2_O and 3% S. HumiAgra was used in the amount of 3 g C kg^−1^ of d.m. soil		
The duration of the experiment	Total: 50 days		
Number repetitions	Vases were with five repetitions per treatment, arranged in random, with complete blocks on tables in the same vegetation hall		
Conditions in the vegetation hall	June–July 2021: day length ranged from 15 h 5 min to 17 h 5 min; the average temperature was 16.5 °C, and the average humidity was 77.5%. Natural light was used, watering up to 60% m.w.c. deionized water		

HAC—hydrolytic acidity, EBC—sum of exchangeable base cations, CEC—cation exchange capacity, ACS—alkaline cation saturation, tot—total, org—organic, av—available.

## Data Availability

Data are available by contacting the authors.

## Supplementary Materials

The supporting information can be downloaded at: https://www.mdpi.com/article/10.3390/ijms24010178/s1.

## Abbreviations

H-HumiAgra; Zm-*Zea mays*; Cr-Cr(VI); SPAD-the leaf greenness index; Org-organotrophic bacteria; Act-actinomycetes; Fun-fungi; CD-colony development index; EP-ecophysiological diversity index; D-chromium uptake by *Zea mays*; TI-tolerance index; TF-translocation index; BF_A_-aerial bioaccumulation index; BF_R_–root bioaccumulation index; BF-accumulation index; IF-index of the influence; HAC-hydrolytic acidity; EBC-sum of exchangeable base cations; CEC-cation exchange capacity; ACS-alkaline cation saturation; C_org_-total organic carbon; N_total_—total nitrogen.

## References

[1] Briffa J., Sinagra E., Blundell R. (2020). Heavy Metal Pollution in the Environment and Their Toxicological Effects on Humans. Heliyon.

[2] Rahman Z., Singh V.P. (2019). The Relative Impact of Toxic Heavy Metals (THMs) (Arsenic (As), Cadmium (Cd), Chromium (Cr)(VI), Mercury (Hg), and Lead (Pb) on the Total Environment: An Overview. Environ. Monit. Assess..

[3] Tchounwou P.B., Yedjou C.G., Patlolla A.K., Sutton D.J., Luch A. (2012). Heavy Metal Toxicity and the Environment. Molecular, Clinical and Environmental Toxicology.

[4] Prasad S., Yadav K.K., Kumar S., Gupta N., Cabral-Pinto M.M.S., Rezania S., Radwan N., Alam J. (2021). Chromium Contamination and Effect on Environmental Health and Its Remediation: A Sustainable Approaches. J. Environ. Manag..

[5] Coetzee J.J., Bansal N., Chirwa E.M.N. (2020). Chromium in Environment, Its Toxic Effect from Chromite-Mining and Ferrochrome Industries, and Its Possible Bioremediation. Expo. Health.

[6] Boros-Lajszner E., Wyszkowska J., Kucharski J. (2021). Phytoremediation of Soil Contaminated with Nickel, Cadmium and Cobalt. Int. J. Phytoremediation.

[7] Kumar S., Prasad S., Yadav K.K., Shrivastava M., Gupta N., Nagar S., Bach Q.-V., Kamyab H., Khan S.A., Yadav S. (2019). Hazardous Heavy Metals Contamination of Vegetables and Food Chain: Role of Sustainable Remediation Approaches—A Review. Environ. Res..

[8] Li Q., Wang Y., Li Y., Li L., Tang M., Hu W., Chen L., Ai S. (2022). Speciation of Heavy Metals in Soils and Their Immobilization at Micro-Scale Interfaces among Diverse Soil Components. Sci. Total Environ..

[9] Alengebawy A., Abdelkhalek S.T., Qureshi S.R., Wang M.-Q. (2021). Heavy Metals and Pesticides Toxicity in Agricultural Soil and Plants: Ecological Risks and Human Health Implications. Toxics.

[10] Zaborowska M., Kucharski J., Wyszkowska J. (2016). Biological Activity of Soil Contaminated with Cobalt, Tin, and Molybdenum. Environ. Monit. Assess..

[11] Zaborowska M., Wyszkowska J., Kucharski J. (2015). Maintenance of Soil Homeostasis under Exposure to Cadmium. Commun. Soil Sci. Plant Anal..

[12] Karimi-Maleh H., Ayati A., Ghanbari S., Orooji Y., Tanhaei B., Karimi F., Alizadeh M., Rouhi J., Fu L., Sillanpää M. (2021). Recent Advances in Removal Techniques of Cr(VI) Toxic Ion from Aqueous Solution: A Comprehensive Review. J. Mol. Liq..

[13] Ukhurebor K.E., Aigbe U.O., Onyancha R.B., Nwankwo W., Osibote O.A., Paumo H.K., Ama O.M., Adetunji C.O., Siloko I.U. (2021). Effect of Hexavalent Chromium on the Environment and Removal Techniques: A Review. J. Environ. Manag..

[14] Sun H., Costa M., Nordberg G.F., Costa M. (2022). Chapter 8—Chromium. Handbook on the Toxicology of Metals.

[15] Jobby R., Jha P., Yadav A.K., Desai N. (2018). Biosorption and Biotransformation of Hexavalent Chromium [Cr(VI)]: A Comprehensive Review. Chemosphere.

[16] Namieśnik J., Rabajczyk A. (2012). Speciation Analysis of Chromium in Environmental Samples. Crit. Rev. Environ. Sci. Technol..

[17] Ertani A., Mietto A., Borin M., Nardi S. (2017). Chromium in Agricultural Soils and Crops: A Review. Water Air Soil Pollut..

[18] Fendorf S., Wielinga B.W., Hansel C.M. (2000). Chromium Transformations in Natural Environments: The Role of Biological and Abiological Processes in Chromium(VI) Reduction. Int. Geol. Rev..

[19] Barnie S., Zhang J., Obeng P.A., Duncan A.E., Adenutsi C.D., Xu L., Chen H. (2021). Mechanism and Multi-Step Kinetic Modelling of Cr(VI) Adsorption, Reduction and Complexation by Humic Acid, Humin and Kerogen from Different Sources. Environ. Sci. Pollut. Res..

[20] Lilli M.A., Nikolaidis N.P., Karatzas G.P., Kalogerakis N. (2019). Identifying the Controlling Mechanism of Geogenic Origin Chromium Release in Soils. J. Hazard. Mater..

[21] Xu T., Jiang X., Tang Y., Zeng Y., Zhang W., Shi B. (2021). Oxidation of Trivalent Chromium Induced by Unsaturated Oils: A Pathway for Hexavalent Chromium Formation in Soil. J. Hazard. Mater..

[22] Bansal N., Coetzee J.J., Chirwa E.M.N. (2019). In Situ Bioremediation of Hexavalent Chromium in Presence of Iron by Dried Sludge Bacteria Exposed to High Chromium Concentration. Ecotoxicol. Environ. Saf..

[23] Shaheen S.M., Tsadilas C.D., Rinklebe J. (2013). A Review of the Distribution Coefficients of Trace Elements in Soils: Influence of Sorption System, Element Characteristics, and Soil Colloidal Properties. Adv. Colloid Interface Sci..

[24] Borah P., Singh P., Rangan L., Karak T., Mitra S. (2018). Mobility, Bioavailability and Ecological Risk Assessment of Cadmium and Chromium in Soils Contaminated by Paper Mill Wastes. Ground Sustain. Dev..

[25] Banks M.K., Schwab A.P., Henderson C. (2006). Leaching and Reduction of Chromium in Soil as Affected by Soil Organic Content and Plants. Chemosphere.

[26] Guo S., Xiao C., Zhou N., Chi R. (2021). Speciation, Toxicity, Microbial Remediation and Phytoremediation of Soil Chromium Contamination. Environ. Chem. Lett..

[27] Mukherjee K., Saha R., Ghosh A., Saha B. (2013). Chromium Removal Technologies. Res. Chem. Intermed..

[28] Dhal B., Thatoi H.N., Das N.N., Pandey B.D. (2013). Chemical and Microbial Remediation of Hexavalent Chromium from Contaminated Soil and Mining/Metallurgical Solid Waste: A Review. J. Hazard. Mater..

[29] Yu X.-Z., Lu C.-J., Tang S., Zhang Q. (2020). Transcriptomic Analysis of Cytochrome P450 Genes and Pathways Involved in Chromium Toxicity in *Oryza sativa*. Ecotoxicology.

[30] Takahashi H., Kopriva S., Giordano M., Saito K., Hell R. (2011). Sulfur Assimilation in Photosynthetic Organisms: Molecular Functions and Regulations of Transporters and Assimilatory Enzymes. Annu. Rev. Plant Biol..

[31] López-Bucio J.S., Ravelo-Ortega G., López-Bucio J. (2022). Chromium in Plant Growth and Development: Toxicity, Tolerance and Hormesis. Environ. Pollut..

[32] Terzi H., Yıldız M. (2021). Proteomic Analysis Reveals the Role of Exogenous Cysteine in Alleviating Chromium Stress in Maize Seedlings. Ecotoxicol. Environ. Saf..

[33] Kotaś J., Stasicka Z. (2000). Chromium Occurrence in the Environment and Methods of Its Speciation. Environ. Pollut..

[34] Saha R., Nandi R., Saha B. (2011). Sources and Toxicity of Hexavalent Chromium. J. Coord. Chem..

[35] Bielicka A., Bojanowska I., Wiśniewski A. (2005). Two Faces of Chromium-Pollutant and Bioelement. Pol. J. Environ. Stud..

[36] Fuge R., Selinus O. (2013). Anthropogenic Sources. Essentials of Medical Geology: Revised Edition.

[37] Wu Q., Mo W., Liu J., Peng S., Li Q., Wan R. (2022). Remediation of High-Concentration Cr(VI)-Contaminated Soils with FeSO_4_ Combined with Biostimulation: Cr(VI) Transformation and Stabilization. J. Hazard. Mater. Adv..

[38] Murthy M.K., Khandayataray P., Padhiary S., Samal D. (2022). A Review on Chromium Health Hazards and Molecular Mechanism of Chromium Bioremediation. Rev. Environ. Health.

[39] Naikoo M.I., Dar M.I., Khan F.A., Raghib F., Rajakaruna N. (2019). Trophic transfer and bioaccumulation of lead along soil–plant–aphid–ladybird food chain. Environ. Sci. Pollut. Res..

[40] Ao M., Chen X., Deng T., Sun S., Tang Y., Morel J.L., Qiu R., Wang S. (2022). Chromium Biogeochemical Behaviour in Soil-Plant Systems and Remediation Strategies: A Critical Review. J. Hazard. Mater..

[41] International Agency for Research on Cancer (1987). Overall Evaluations of Carcinogenicity: An Updating of IARC Monographs Volumes 1 to 42.

[42] Agency for Toxic Substances and Disease Registry, USA Substance Priority List|ATSDR. https://www.atsdr.cdc.gov/spl/index.html.

[43] Arshad M., Khan A.H.A., Hussain I., Badar-uz-Zaman, Anees M., Iqbal M., Soja G., Linde C., Yousaf S. (2017). The Reduction of Chromium (VI) Phytotoxicity and Phytoavailability to Wheat (*Triticum aestivum* L.) Using Biochar and Bacteria. Appl. Soil Ecol..

[44] Katsayal B.S., Sallau A.B., Muhammad A. (2022). Kinetics and Thermodynamics of Cr (VI) Reduction by Tamarindus Indica Methanol Leaves Extract under Optimized Reaction Conditions. Beni-Suef Univ. J. Basic Appl. Sci..

[45] Zanganeh F., Heidari A., Sepehr A., Rohani A. (2022). Bioaugmentation and Bioaugmentation–Assisted Phytoremediation of Heavy Metal Contaminated Soil by a Synergistic Effect of Cyanobacteria Inoculation, Biochar, and Purslane (*Portulaca oleracea* L.). Environ. Sci. Pollut. Res..

[46] Wyszkowska J., Borowik A., Zaborowska M., Kucharski J. (2022). Evaluation of the Usefulness of Sorbents in the Remediation of Soil Exposed to the Pressure of Cadmium and Cobalt. Materials.

[47] Wyszkowska J., Borowik A., Zaborowska M., Kucharski J. (2022). Mitigation of the Adverse Impact of Copper, Nickel, and Zinc on Soil Microorganisms and Enzymes by Mineral Sorbents. Materials.

[48] Azeez N.A., Dash S.S., Gummadi S.N., Deepa V.S. (2021). Nano-Remediation of Toxic Heavy Metal Contamination: Hexavalent Chromium [Cr(VI)]. Chemosphere.

[49] Zaborowska M., Wyszkowska J., Kucharski J. (2019). Biochemical Activity of Soil Contaminated with BPS, Bioaugmented with a Mould Fungi Consortium and a Bacteria Consortium. Environ. Sci. Pollut. Res..

[50] Wyszkowska J., Boros-Lajszner E., Borowik A., Kucharski J. (2022). The Role of Cellulose in Microbial Diversity Changes in the Soil Contaminated with Cadmium. Sustainability.

[51] Ju L., Jiao Z., Ge S., Zhan W., Liu Y., Ren Q., Liao Q., Yang Z., Wang Y. (2022). Formation, Stability and Mobility of Soluble Cr(III) during Cr(VI) Reduction by *Pannonibacter phragmitetus* BB. Environ. Technol. Innov..

[52] Galani A., Mamais D., Noutsopoulos C., Anastopoulou P., Varouxaki A. (2022). Biotic and Abiotic Biostimulation for the Reduction of Hexavalent Chromium in Contaminated Aquifers. Water.

[53] Erisman J.W., van Eekeren N., De Wit J., Koopmans C.J., Cuijpers W.J.M., Oerlemans N., Koks B. (2016). Agriculture and Biodiversity: A Better Balance Benefits Both. AIMS Agric. Food.

[54] Nowicka B. (2022). Heavy metal–induced stress in eukaryotic algae—mechanisms of heavy metal toxicity and tolerance with particular emphasis on oxidative stress in exposed cells and the role of antioxidant response. Environ. Sci. Pollut. Res..

[55] Mohammed B., Mohammed T., M’hammed E., Tarik A. (2021). Physiological and Physico-Chemical Study of the Effect of Chromium VI on the Nutritional Quality of Maize (*Zea mays* L). Procedia Comput. Sci..

[56] Polti M.A., Atjián M.C., Amoroso M.J., Abate C.M. (2011). Soil Chromium Bioremediation: Synergic Activity of Actinobacteria and Plants. Int. Biodeterior. Biodegrad..

[57] Yang S., Ulhassan Z., Shah A.M., Khan A.R., Azhar W., Hamid Y., Hussain S., Sheteiwy M.S., Salam A., Zhou W. (2021). Salicylic Acid Underpins Silicon in Ameliorating Chromium Toxicity in Rice by Modulating Antioxidant Defense, Ion Homeostasis and Cellular Ultrastructure. Plant Physiol. Biochem..

[58] Wakeel A., Xu M. (2020). Chromium Morpho-Phytotoxicity. Plants.

[59] Stambulska U.Y., Bayliak M.M., Lushchak V.I. (2018). Chromium(VI) Toxicity in Legume Plants: Modulation Effects of Rhizobial Symbiosis. BioMed Res. Int..

[60] Sharma D.C., Sharma C.P., Tripathi R.D. (2003). Phytotoxic Lesions of Chromium in Maize. Chemosphere.

[61] Ulhassan Z., Khan I., Hussain M., Khan A.R., Hamid Y., Hussain S., Allakhverdiev S.I., Zhou W. (2022). Efficacy of Metallic Nanoparticles in Attenuating the Accumulation and Toxicity of Chromium in Plants: Current Knowledge and Future Perspectives. Environ. Pollut..

[62] Rodriguez E., Santos C., Azevedo R., Moutinho-Pereira J., Correia C., Dias M.C. (2012). Chromium (VI) Induces Toxicity at Different Photosynthetic Levels in Pea. Plant Physiol. Biochem..

[63] Sharma A., Kapoor D., Wang J., Shahzad B., Kumar V., Bali A.S., Jasrotia S., Zheng B., Yuan H., Yan D. (2020). Chromium Bioaccumulation and Its Impacts on Plants: An Overview. Plants.

[64] Singh H.P., Mahajan P., Kaur S., Batish D.R., Kohli R.K. (2013). Chromium Toxicity and Tolerance in Plants. Environ. Chem. Lett..

[65] Hamilton E.M., Young S.D., Bailey E.H., Humphrey O.S., Watts M.J. (2020). Assessment of Chromium Species Dynamics in Root Solutions Using Isotope Tracers. J. Trace Elem. Med. Biol..

[66] Afshan S., Ali S., Bharwana S.A., Rizwan M., Farid M., Abbas F., Ibrahim M., Mehmood M.A., Abbasi G.H. (2015). Citric Acid Enhances the Phytoextraction of Chromium, Plant Growth, and Photosynthesis by Alleviating the Oxidative Damages in *Brassica napus* L.. Environ Sci. Pollut. Res..

[67] Gondar D., López R., Fiol S., Antelo J.M., Arce F. (2006). Cadmium, Lead, and Copper Binding to Humic Acid and Fulvic Acid Extracted from an Ombrotrophic Peat Bog. Geoderma.

[68] Garcia-Mina J.M. (2006). Stability, Solubility and Maximum Metal Binding Capacity in Metal–Humic Complexes Involving Humic Substances Extracted from Peat and Organic Compost. Org. Geochem..

[69] Tang X., Huang Y., Li Y., Wang L., Pei X., Zhou D., He P., Hughes S.S. (2021). Study on Detoxification and Removal Mechanisms of Hexavalent Chromium by Microorganisms. Ecotoxicol. Environ. Saf..

[70] Lowe K.L., Straube W., Little B., Jones-Meehan J. (2003). Aerobic and Anaerobic Reduction of Cr(VI) by *Shewanella Oneidensis* Effects of Cationic Metals, Sorbing Agents and Mixed Microbial Cultures. Acta Biotechnol..

[71] Singh P., Itankar N., Patil Y. (2021). Biomanagement of Hexavalent Chromium: Current Trends and Promising Perspectives. J. Environ. Manag..

[72] Villacís-García M., Villalobos M., Gutiérrez-Ruiz M. (2015). Optimizing the Use of Natural and Synthetic Magnetites with Very Small Amounts of Coarse Fe(0) Particles for Reduction of Aqueous Cr(VI). J. Hazard. Mater..

[73] Uddin M.J., Jeong Y.-K., Lee W. (2021). Microbial Fuel Cells for Bioelectricity Generation through Reduction of Hexavalent Chromium in Wastewater: A Review. Int. J. Hydrogen Energy.

[74] Xu T., Nan F., Jiang X., Tang Y., Zeng Y., Zhang W., Shi B. (2020). Effect of Soil PH on the Transport, Fractionation, and Oxidation of Chromium(III). Ecotoxicol. Environ. Saf..

[75] Löv Å., Sjöstedt C., Larsbo M., Persson I., Gustafsson J.P., Cornelis G., Kleja D.B. (2017). Solubility and Transport of Cr(III) in a Historically Contaminated Soil—Evidence of a Rapidly Reacting Dimeric Cr(III) Organic Matter Complex. Chemosphere.

[76] Wyszkowska J., Kucharski J., Jastrzębska E., Hłasko A. (2001). The Biological Properties of Soil as Influenced by Chromium Contamination. Pol. J. Environ. Stud..

[77] Mushtaq Z., Liaquat M., Nazir A., Liaquat R., Iftikhar H., Anwar W., Itrat N. (2022). Potential of Plant Growth Promoting Rhizobacteria to Mitigate Chromium Contamination. Environ. Technol. Innov..

[78] Wang C., Cui Y. (2019). Recognition of a New Cr(VI)-Reducing Strain and Study of the Potential Capacity for Reduction of Cr(VI) of the Strain. BioMed Res..

[79] Ramírez-Díaz M.I., Díaz-Pérez C., Vargas E., Riveros-Rosas H., Campos-García J., Cervantes C. (2008). Mechanisms of Bacterial Resistance to Chromium Compounds. Biometals.

[80] Castro C., Urbieta M.S., Plaza Cazón J., Donati E.R. (2019). Metal Biorecovery and Bioremediation: Whether or Not Thermophilic Are Better than Mesophilic Microorganisms. Bioresour. Technol..

[81] Kathiravan M.N., Karthick R., Muthukumar K. (2011). Ex Situ Bioremediation of Cr(VI) Contaminated Soil by *Bacillus* Sp.: Batch and Continuous Studies. Chem. Eng. J..

[82] Kalola V., Desai C. (2020). Biosorption of Cr(VI) by *Halomonas* Sp. DK4, a Halotolerant Bacterium Isolated from Chrome Electroplating Sludge. Environ. Sci. Pollut. Res. Int..

[83] Jobby R., Jha P., Gupta A., Gupte A., Desai N. (2019). Biotransformation of Chromium by Root Nodule Bacteria *Sinorhizobium* Sp. SAR1. PLoS ONE.

[84] Campitelli P.A., Velasco M.I., Ceppi S.B. (2006). Chemical and Physicochemical Characteristics of Humic Acids Extracted from Compost, Soil and Amended Soil. Talanta.

[85] Borges F., Guimarães C., Lima J.L.F.C., Pinto I., Reis S. (2005). Potentiometric Studies on the Complexation of Copper(II) by Phenolic Acids as Discrete Ligand Models of Humic Substances. Talanta.

[86] Wyszkowska J., Borowik A., Kucharski J., Baćmaga M., Tomkiel M., Boros-Lajszner E. (2013). The Effect of Organic Fertilizers on the Biochemical Properties of Soil Contaminated with Zinc. Plant Soil Environ..

[87] Zaborowska M., Wyszkowska J., Borowik A., Kucharski J. (2022). Effect of Separate and Combined Toxicity of Bisphenol A and Zinc on the Soil Microbiome. Int. J. Mol. Sci..

[88] Polti M.A., García R.O., Amoroso M.J., Abate C.M. (2009). Bioremediation of Chromium(VI) Contaminated Soil by *Streptomyces* Sp. MC1. J. Basic Microbiol..

[89] Laxman R.S., More S. (2002). Reduction of Hexavalent Chromium by Streptomyces Griseus. Miner. Eng..

[90] Polti M.A., Amoroso M.J., Abate C.M. (2011). Intracellular Chromium Accumulation by *Streptomyces* Sp. MC1. Water Air Soil Pollut..

[91] Fernández P.M., Viñarta S.C., Bernal A.R., Cruz E.L., Figueroa L.I.C. (2018). Bioremediation Strategies for Chromium Removal: Current Research, Scale-up Approach and Future Perspectives. Chemosphere.

[92] Vimala R., Das N. (2011). Mechanism of Cd(II) Adsorption by Macrofungus *Pleurotus platypus*. J. Environ. Sci..

[93] Canton G.C., Bertolazi A.A., Cogo A.J.D., Eutrópio F.J., Melo J., de Souza S.B., Krohling C.A., Campostrini E., da Silva A.G., Façanha A.R. (2016). Biochemical and Ecophysiological Responses to Manganese Stress by Ectomycorrhizal Fungus Pisolithus Tinctorius and in Association with Eucalyptus Grandis. Mycorrhiza.

[94] Salam M., Varma A. (2019). Bacterial Community Structure in Soils Contaminated with Electronic Waste Pollutants from Delhi NCR, India. Electron. J. Biotechnol..

[95] Delgado-Baquerizo M., Oliverio A.M., Brewer T.E., Benavent-González A., Eldridge D.J., Bardgett R.D., Maestre F.T., Singh B.K., Fierer N. (2018). A Global Atlas of the Dominant Bacteria Found in Soil. Science.

[96] Zaborowska M., Wyszkowska J., Borowik A. (2020). Soil Microbiome Response to Contamination with Bisphenol A, Bisphenol F and Bisphenol S. Int. J. Mol. Sci..

[97] Chen Y., Jiang Y., Huang H., Mou L., Ru J., Zhao J., Xiao S. (2018). Long-Term and High-Concentration Heavy-Metal Contamination Strongly Influences the Microbiome and Functional Genes in Yellow River Sediments. Sci. Total Environ..

[98] Thatoi H., Das S., Mishra J., Rath B.P., Das N. (2014). Bacterial Chromate Reductase, a Potential Enzyme for Bioremediation of Hexavalent Chromium: A Review. J. Environ. Manag..

[99] Karthik C., Barathi S., Pugazhendhi A., Ramkumar V.S., Thi N.B.D., Arulselvi P.I. (2017). Evaluation of Cr(VI) Reduction Mechanism and Removal by Cellulosimicrobium Funkei Strain AR8, a Novel Haloalkaliphilic Bacterium. J. Hazard. Mater..

[100] Bharagava R.N., Mishra S. (2018). Hexavalent Chromium Reduction Potential of *Cellulosimicrobium* Sp. Isolated from Common Effluent Treatment Plant of Tannery Industries. Ecotoxicol. Environ. Saf..

[101] Sathishkumar K., Murugan K., Benelli G., Higuchi A., Rajasekar A. (2017). Bioreduction of Hexavalent Chromium by *Pseudomonas stutzeri* L1 and *Acinetobacter naumannii* L2. Ann. Microbiol..

[102] Banerjee S., Kamila B., Barman S., Joshi S.R., Mandal T., Halder G. (2019). Interlining Cr(VI) Remediation Mechanism by a Novel Bacterium *Pseudomonas Brenneri* Isolated from Coalmine Wastewater. J. Environ. Manag..

[103] Zhang Q., Amor K., Galer S.J.G., Thompson I., Porcelli D. (2019). Using Stable Isotope Fractionation Factors to Identify Cr(VI) Reduction Pathways: Metal-Mineral-Microbe Interactions. Water Res..

[104] Chen J.M., Hao O.J. (1998). Microbial Chromium (VI) Reduction. Crit. Rev. Environ. Sci. Technol..

[105] He Y., Dong L., Zhou S., Jia Y., Gu R., Bai Q., Gao J., Li Y., Xiao H. (2018). Chromium Resistance Characteristics of Cr(VI) Resistance Genes ChrA and ChrB in *Serratia* Sp. S2. Ecotoxicol. Environ. Saf..

[106] Viti C., Marchi E., Decorosi F., Giovannetti L. (2014). Molecular Mechanisms of Cr(VI) Resistance in Bacteria and Fungi. FEMS Microbiol. Rev..

[107] Dong L., Zhou S., He Y., Jia Y., Bai Q., Deng P., Gao J., Li Y., Xiao H. (2018). Analysis of the Genome and Chromium Metabolism-Related Genes of *Serratia* Sp. S2. Appl. Biochem. Biotechnol..

[108] Li X., Yin Q., Gu R., Li M., Yan J., Liu Y., Qiu Y., Bai Q., Li Y., Ji Y. (2021). Effects of Exogenous Sulfate on the Chromium(VI) Metabolism of Chromium(VI)-Resistant Engineered Strains. Ecotoxicol. Environ. Saf..

[109] Shaw D.R., Dussan J. (2018). Transcriptional Analysis and Molecular Dynamics Simulations Reveal the Mechanism of Toxic Metals Removal and Efflux Pumps in *Lysinibacillus Sphaericus* OT4b.31. Int. Biodeterior. Biodegrad..

[110] Li Y., Li Q., Fengying Y., Bao J., Hu Z., Zhu W., Zhao Y., Lin Z., Dong Q. (2015). Chromium (VI) Detoxification by Oxidation and Flocculation of Exopolysaccharides from *Arthrobacter* Sp. B4. Int. J. Biol. Macromol..

[111] Pi S., Li A., Qiu J., Feng L., Zhou L., Zhao H.-P., Ma F. (2021). Enhanced Recovery of Hexavalent Chromium by Remodeling Extracellular Polymeric Substances through Engineering *Agrobacterium Tumefaciens* F2. J. Clean. Prod..

[112] Yu C., Tang X., Li L.-S., Chai X.-L., Xiao R., Wu D., Tang C.-J., Chai L.-Y. (2019). The Long-Term Effects of Hexavalent Chromium on Anaerobic Ammonium Oxidation Process: Performance Inhibition, Hexavalent Chromium Reduction and Unexpected Nitrite Oxidation. Bioresour. Technol..

[113] Şahin Y., Öztürk A. (2005). Biosorption of Chromium(VI) Ions from Aqueous Solution by the Bacterium *Bacillus thuringiensis*. Proc. Biochem..

[114] Kiliç N.K., Stensballe A., Otzen D.E., Dönmez G. (2010). Proteomic Changes in Response to Chromium(VI) Toxicity in *Pseudomonas aeruginosa*. Bioresour. Technol..

[115] Wang B., Zhu S., Li W., Tang Q., Luo H. (2021). Effects of Chromium Stress on the Rhizosphere Microbial Community Composition of *Cyperus alternifolius*. Ecotoxicol. Environ. Saf..

[116] Benila Smily J.R.M., Sumithra P.A. (2017). Optimization of Chromium Biosorption by Fungal Adsorbent, *Trichoderma* Sp. BSCR02 and Its Desorption Studies. HAYATI J. Biosci..

[117] Saranya N., Suganya E., Selvaraju N., Senthilkumar S., Sivasubramanian V., Sivakumar P., Raja S. (2020). 3-Level Box–Behnkenoptimization of Hexavalent Chromium Reduction by Chromate Resistant *Trichoderma asperellum* Cells from Simulated and Industrial Effluent. Environ. Technol. Innov..

[118] Vankar P.S., Bajpai D. (2008). Phyto-Remediation of Chrome-VI of Tannery Effluent by *Trichoderma* Species. Desalination.

[119] Arévalo-Rangel D.L., Cárdenas-González J.F., Martínez-Juárez V.M., Acosta-Rodríguez I. (2013). Hexavalent Chromate Reductase Activity in Cell Free Extracts of *Penicillium* Sp.. Bioinorg. Chem. Appl..

[120] Ahemad M. (2014). Bacterial Mechanisms for Cr(VI) Resistance and Reduction: An Overview and Recent Advances. Folia Microbiol..

[121] Bao S., Mu J., Yin P., Chen H., Zhou S. (2022). Exploration of Anti-Chromium Mechanism of Marine *Penicillium janthinellum* P1 through Combinatorial Transcriptomic Analysis and WGCNA. Ecotoxicol. Environ. Saf..

[122] IUSS Working Group WRB (2015). World Reference Base for Soil Resources 2014, Update 2015. International Soil Classification System for Naming Soils and Creating Legends for Soil Maps.

[123] (2021). OECD-FAO Agricultural Outlook 2021–2030.

[124] Erenstein O., Chamberlin J., Sonder K. (2021). Estimating the Global Number and Distribution of Maize and Wheat Farms. Glob. Food Secur..

[125] AL-Huqail A.A., El-Bondkly A.M.A. (2022). Improvement of *Zea mays* L. Growth Parameters under Chromium and Arsenic Stress by the Heavy Metal-Resistant *Streptomyces* Sp. NRC21696. Int. J. Environ. Sci. Technol..

[126] Meers E., Van Slycken S., Adriaensen K., Ruttens A., Vangronsveld J., Du Laing G., Witters N., Thewys T., Tack F.M.G. (2010). The Use of Bio-Energy Crops (*Zea mays*) for ‘Phytoattenuation’ of Heavy Metals on Moderately Contaminated Soils: A Field Experiment. Chemosphere.

[127] Morales-Máximo C.N., López-Sosa L.B., Rutiaga-Quiñones J.G., Corral-Huacuz J.C., Aguilera-Mandujano A., Pintor-Ibarra L.F., López-Miranda A., Delgado-Domínguez S.N., Rodríguez-Magallón M.d.C., Morales-Máximo M. (2022). Characterization of Agricultural Residues of *Zea mays* for Their Application as Solid Biofuel: Case Study in San Francisco Pichátaro, Michoacán, Mexico. Energies.

[128] Boros-Lajszner E., Wyszkowska J., Borowik A., Kucharski J. (2021). Energetic Value of *Elymus elongatus* L. and *Zea mays* L. Grown on Soil Polluted with Ni^2+^, Co^2+^, Cd^2+^, and Sensitivity of Rhizospheric Bacteria to Heavy Metals. Energies.

[129] Ali H., Khan E., Ilahi I. (2019). Environmental Chemistry and Ecotoxicology of Hazardous Heavy Metals: Environmental Persistence, Toxicity, and Bioaccumulation. J. Chem..

[130] Fu Z., Guo W., Dang Z., Hu Q., Wu F., Feng C., Zhao X., Meng W., Xing B., Giesy J.P. (2017). Refocusing on Nonpriority Toxic Metals in the Aquatic Environment in China. Environ. Sci. Technol..

[131] Bunt J.S., Rovira A.D. (1955). Microbiological Studies of Some Subantarctic Soils. J. Soil Sci..

[132] Parkinson D., Gray T.R.G., Williams S.T. (1971). Methods for Studying the Ecology of Soil Microorganisms.

[133] Martin J.P. (1950). Use of Acid, Rose Bengal, and Streptomycin in the Plate Method for Estimating Soil Fungi. Soil Sci..

[134] Borowik A., Wyszkowska J., Kucharski M., Kucharski J. (2019). Implications of Soil Pollution with Diesel Oil and BP Petroleum with ACTIVE Technology for Soil Health. Int. J. Environ. Res. Public Health.

[135] Ferris M.J., Muyzer G., Ward D.M. (1996). Denaturing Gradient Gel Electrophoresis Profiles of 16S RRNA-Defined Populations Inhabiting a Hot Spring Microbial Mat Community. Appl. Environ. Microbiol..

[136] Wyszkowska J., Borowik A., Kucharski J. (2022). The Role of Grass Compost and *Zea Mays* in Alleviating Toxic Effects of Tetracycline on the Soil Bacteria Community. Int. J. Environ. Res. Public Health.

[137] Borowik A., Wyszkowska J., Wyszkowski M. (2017). Resistance of Aerobic Microorganisms and Soil Enzyme Response to Soil Contamination with Ekodiesel Ultra Fuel. Environ. Sci. Pollut. Res..

[138] (2013). Soil Quality—Determination of Cadmium, Chromium, Cobalt, Copper, Lead, Manganese, Nickel and Zinc in Aqua Regia Extracts of Soil—Flame and Electrothermal Atomic Absorption Spectrometric Methods.

[139] (2002). Polish Committee for Standardization. Soil Quality—Extraction of Trace Elements Soluble in Aqua Regia.

[140] De Leij F.A.A.M., Whipps J.M., Lynch J.M. (1993). The Use of Colony Development for the Characterization of Bacterial Communities in Soil and on Roots. Microb. Ecol..

[141] Borowik A., Wyszkowska J., Oszust K. (2017). Functional Diversity of Fungal Communities in Soil Contaminated with Diesel Oil. Front. Microbiol..

[142] (2017). TIBCO Software Inc Statistica (Data Analysis Software System), Version 13. http://statistica.io.

[143] RStudio Team (2019). RStudio: Integrated Development for R.

[144] R Core Team (2019). R: A Language and Environment for Statistical Computing.

[145] Warnes G.R., Bolker B., Bonebakker L., Gentleman R., Huber W., Liaw A., Lumley T., Maechler M., Magnusson M., Moeller S. (2020). Gplots: Various R Programming Tools for Plotting Data. R Package Version 2.17.0. https://cran.r-project.org/package=gplots.

[146] Parks D.H., Tyson G.W., Hugenholtz P., Beiko R.G. (2014). STAMP: Statistical Analysis of Taxonomic and Functional Profiles. Bioinformatics.

[147] Heberle H., Meirelles G.V., da Silva F.R., Telles G.P., Minghim R. (2015). InteractiVenn: A Web-Based Tool for the Analysis of Sets through Venn Diagrams. BMC Bioinform..

