# Supplementary material for: Sensitivity of Zea mays and Soil Microorganisms to the Toxic Effect of Chromium (VI)
Source: Int J Mol Sci. 2022 Dec 22;24(1):178. doi: 10.3390/ijms24010178 (PMC9820705; doi:10.3390/ijms24010178)
Supplement: Supplementary file 1 [file ijms-24-00178-s001.zip › ijms-2109139-supplementary.pdf]

International Journal of
Molecular Sciences

Supplementary Materials

Sensitivity of *Zea mays* and soil microorganisms to the toxic effect of chromium (VI)

Jadwiga Wyszkowska *, Agata Borowik, Magdalena Zaborowska and Jan Kucharski

Department of Soil Science and Microbiology, Faculty of Agriculture and Forestry, University of Warmia and Mazury in Olsztyn, 10-727 Olsztyn, Poland

* Correspondence: jadwiga.wyszkowska@uwm.edu.pl

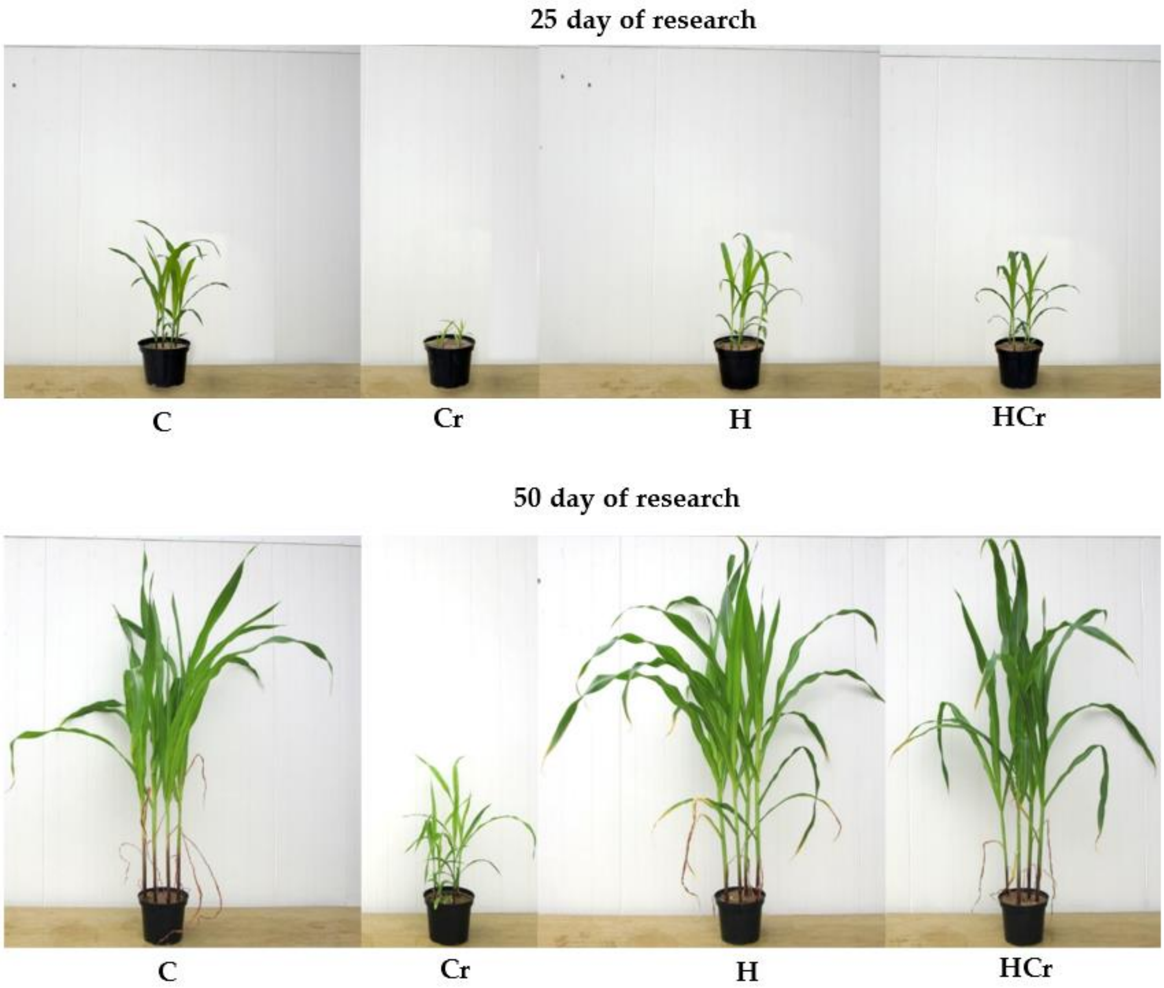

Figure S1. *Zea mays* in the 25th and 50th day of research

Table S1. The number of microorganisms, 10^n cfu kg^{-1} d.m. of soil

**Dose of Cr(VI), mg kg^{-1} d.m. of soil**	**Soil use**			
	**Unsown**		**Sown with *Zea mays***	
	**Analysis day**			
	**25**	**50**	**25**	**50**
**Organotrophic bacteria, 10^9**				
Control				
0	18.340^{de}	14.938^{ef}	35.496^{b}	34.559^{b}
60	7.148^{gh}	2.909^{h}	10.452^{fg}	19.966^{d}
HumiAgra (H)				
0	32.932^{b}	21.199^{d}	35.742^{b}	44.173^{a}
60	13.656^{f}	11.142^{fg}	14.445^{ef}	26.277^{c}
**Actinomyces, 10^9**				
Control				
0	11.142^{hi}	19.326^{ef}	32.390^{b}	27.559^{c}
60	3.648^{j}	12.325^{gh}	17.255^{f}	13.952^{g}
HumiAgra				
0	22.382^{d}	32.193^{b}	36.482^{a}	32.932^{b}
60	9.022^{i}	13.508^{gh}	21.100^{de}	13.656^{gh}
**Fungi, 10^7**				
Control				
0	15.776^{g}	19.326^{c-f}	17.452^{e-g}	21.692^{bc}
60	10.994*i*	15.529*gh*	11.684*i*	16.565*fg*
HumiAgra				
0	19.966^{c-e}	20.854^{b-d}	25.439^{a}	23.565^{ab}
60	12.473^{hi}	15.529^{gh}	18.290^{d-g}	19.473^{c-f}

Homogeneous groups within each microbial group are marked with identical letters (a-j).

Table S2. Colony Development Index (CD) of microorganisms

Dose of Cr(VI), mg kg^{-1} d.m. of soil	Soil use			
	Unsown		Sown with *Zea mays*	
	Analysis day			
	25	50	25	50
**Organotrophic bacteria, 10^9**				
Control				
0	29.522^{a-c}	27.727^{a-d}	29.291*a-c*	28.448^{a-d}
60	31.096^{ab}	26.742^{b-d}	27.016^{b-d}	28.281^{a-d}
HumiAgra				
0	31.434^{a}	31.788^{a}	31.488^{a}	29.638^{a-c}
60	24.767^{d}	26.387^{cd}	25.808^{cd}	28.321^{a-d}
**Actinomyces, 10^9**				
Control				
0	26.555^{bc}	20.448^{f-h}	20.731^{f-h}	30.156^{a}
60	24.535^{c-e}	17.899^{hi}	19.109^{g-i}	24.756^{b-d}
HumiAgra				
0	27.618^{ab}	20.998^{fg}	22.697^{d-f}	21.232^{fg}
60	17.320^{i}	21.534^{e-g}	19.118^{g-i}	19.980^{f-i}
**Fungi, 10^7**				
Control				
0	27.726^{j}	30.735^{i}	30.991^{hi}	33.018^{fg}
60	33.974^{d-f}	36.871^{b}	34.906^{c-e}	37.056^{b}
HumiAgra				
0	30.311^{i}	35.198^{b-d}	31.930^{g-i}	33.096^{e-g}
60	32.811^{f-h}	39.014^{a}	39.007^{a}	35.851^{bc}

Homogeneous groups within each microbial group are marked with identical letters (a-j).

Table S3. Index of the ecophysiological diversity (EP) of microorganisms

Dose of Cr(VI), mg kg^{-1} d.m. of soil	Soil use			
	Unsown		Sown with *Zea mays*	
	Analysis day			
	25	50	25	50
**Organotrophic bacteria, 10^9**				
Control				
0	0.837^{b-d}	0.776^{d}	0.887^{a-c}	0.833^{b-d}
60	0.839^{b-d}	0.892^{ab}	0.880^{bc}	0.827^{cd}
HumiAgra (H)				
0	0.854^{bc}	0.858^{bc}	0.893^{ab}	0.699^{e}
60	0.872^{bc}	0.842^{bc}	0.947^{a}	0.876^{bc}
**Actinomyces, 10^9**				
Control				
0	0.777^{cd}	0.865^{ab}	0.810^{b-d}	0.816^{b-d}
60	0.677^{e}	0.885^{ab}	0.761^{cd}	0.886^{ab}
HumiAgra				
0	0.753^{de}	0.901^{a}	0.893^{ab}	0.908^{a}
60	0.592^{f}	0.873^{ab}	0.841^{a-c}	0.753^{de}
**Fungi, 10^7**				
Control				
0	0.736^{ab}	0.614^{g}	0.708^{bc}	0.673^{d-f}
60	0.649^{e-g}	0.597^{gh}	0.626^{fg}	0.546^{hi}
HumiAgra				
0	0.705^{b-d}	0.609^{g}	0.705^{b-d}	0.783^{a}
60	0.601^{gh}	0.530^{i}	0.509^{i}	0.625^{fg}

Homogeneous groups within each microbial group are marked with identical letters (a-i).
